# *Francisella novicida* Two-Component System Response Regulator BfpR Modulates iglC Gene Expression, Antimicrobial Peptide Resistance, and Biofilm Production

**DOI:** 10.3389/fcimb.2020.00082

**Published:** 2020-03-13

**Authors:** Scott N. Dean, Morgan E. Milton, John Cavanagh, Monique L. van Hoek

**Affiliations:** ^1^National Center for Biodefense and Infectious Diseases, and School of Systems Biology, George Mason University, Manassas, VA, United States; ^2^Department of Biochemistry and Molecular Biology, The Brody School of Medicine, East Carolina University, Greenville, NC, United States

**Keywords:** *Francisella*, response regulator, two component systems (TCSs), biofilm, antimicrobial peptide resistance

## Abstract

Response regulators are a critical part of the two-component system of gene expression regulation in bacteria, transferring a signal from a sensor kinase into DNA binding activity resulting in alteration of gene expression. In this study, we investigated a previously uncharacterized response regulator in *Francisella novicida*, FTN_1452 that we have named BfpR (Biofilm-regulating *Francisella* protein Regulator, *FTN_1452*). In contrast to another Francisella response regulator, QseB/PmrA, BfpR appears to be a negative regulator of biofilm production, and also a positive regulator of antimicrobial peptide resistance in this bacterium. The protein was crystallized and X-ray crystallography studies produced a 1.8 Å structure of the BfpR N-terminal receiver domain revealing interesting insight into its potential interaction with the sensor kinase. Structural analysis of BfpR places it in the OmpR/PhoP family of bacterial response regulators along with WalR and ResD. Proteomic and transcriptomic analyses suggest that BfpR overexpression affects expression of the critical *Francisella* virulence factor iglC, as well as other proteins in the bacterium. We demonstrate that mutation of *bfpR* is associated with an antimicrobial peptide resistance phenotype, a phenotype also associated with other response regulators, for the human cathelicidin peptide LL-37 and a sheep antimicrobial peptide SMAP-29. *F. novicida* with mutated *bfpR* replicated better than WT in intracellular infection assays in human-derived macrophages suggesting that the down-regulation of iglC expression in *bfpR* mutant may enable this intracellular replication to occur. Response regulators have been shown to play important roles in the regulation of bacterial biofilm production. We demonstrate that *F. novicida* biofilm formation was highly increased in the *bfpR* mutant, corresponding to altered glycogen synthesis. Waxworm infection experiments suggest a role of BfpR as a negative modulator of iglC expression with de-repression by Mg^2+^. In this study, we find that the response regulator BfpR may be a negative regulator of biofilm formation, and a positive regulator of antimicrobial peptide resistance in *F. novicida*.

## Introduction

*Francisella* are Gram-negative, fastidious bacteria found primarily in the Northern Hemisphere. The genus is composed of several species, the most notable being the highly virulent *Francisella tularensis*, the causative agent of tularemia. Cases of tularemia in the United States are rare and recently increasing, and interest in *Francisella*-related research is high (Dennis et al., 2001; Oyston et al., 2004). This is due to recent outbreaks of tularemia around the world and the fact that *F. tularensis* is classified as a Category A Biological threat Agent. The less-virulent environmental strain, *F. novicida*, is used as a model for *Francisella* studies due to its high genetic similarity (>97%) to *F. tularensis*, genetic tractability, availability of transposon mutant library, and its biosafety level 2 status due to lack of infectivity for humans (Gallagher et al., 2007; Larsson et al., 2009; Enstrom et al., 2012; Kingry and Petersen, 2014).

*F. novicida* has been shown to form biofilms *in vitro* (Dean et al., 2009; Durham-Colleran et al., 2010; Margolis et al., 2010; Verhoeven et al., 2010; van Hoek, 2013). Biofilms play an important role in bacterial environmental survival and are often involved in pathogenicity and antimicrobial resistance. In *Pseudomonas, Salmonella*, and many other Gram-negative bacteria, biofilm formation is under the control of two-component systems. Two-component systems are ubiquitous bacterial communication models and are typically formed from a membrane bound sensor histidine kinase and a DNA-binding response regulator (Stock et al., 2000; van Hoek et al., 2019). An extracellular signal is detected by the sensor kinase, resulting in autophosphorylation of the sensor kinase. The phosphate group is then transferred to the response regulator. Upon phosphorylation, the response regulator changes from an “inactive” to “active” state. The activated response regulator then dictates cellular responses through transcriptional regulation. Response regulators are transcription factors composed of two domains, a receiver (REC) domain which is connected to a DNA-binding domain through a flexible linker. On a molecular level, the response regulator propagates the signal by accepting the phosphate onto a conserved aspartate located within the REC domain. Activation of the response regulator often causes the REC domain to form a dimer, bringing the DNA-binding domains into proximity (Gao and Stock, 2009). Dimer formation positions the two DNA-binding domains to better bind the two half sites of the response regulator's cognate promoter. Understanding the structure and function of response regulators is important for comprehending bacterial gene expression, and could play a role in the development of new antimicrobial therapies.

**Figure 1:**
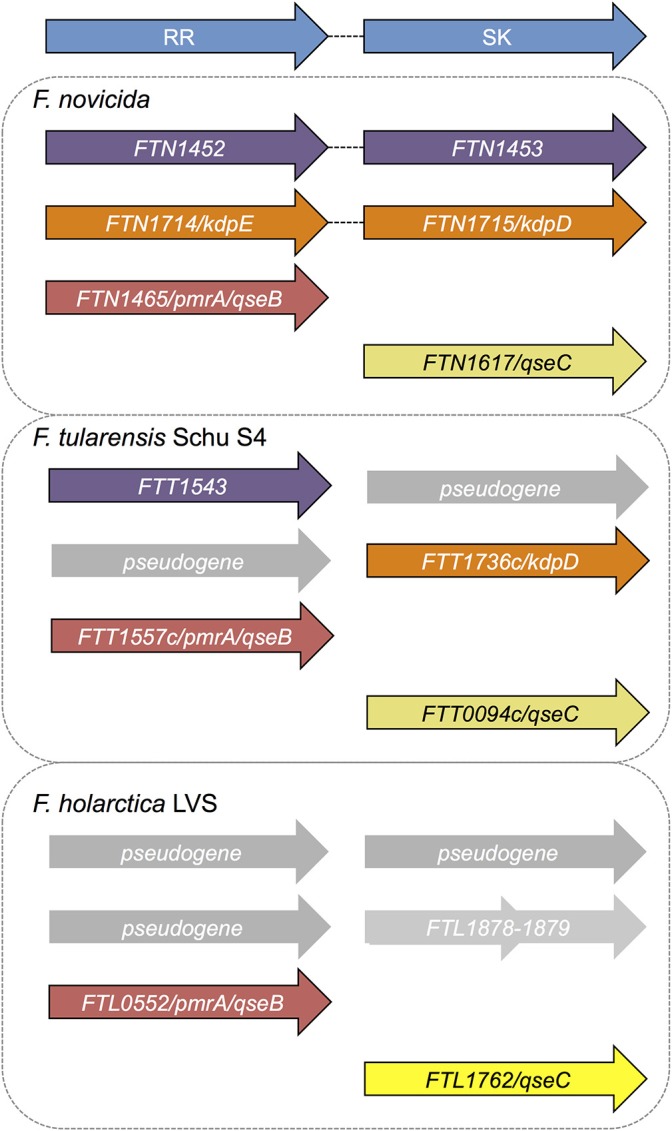
Two component systems annotated or predicted in *Francisella* species. The fully virulent *F. tularensis Schu* S4 genome is annotated to contain two sensor histidine kinases and two response regulators, none of which are within intact (paired) two component systems. The two component system genes previously identified in *F. tularensis Schu* S4 include two sensor kinases FTT_0094c (*qseC*) and FTT_1736c (*kdpD*) and two response regulators FTT_1543 (*bfpR*) and FTT_1557c (*qseB/pmrA*), all of which are considered to be “orphan” or not paired in a canonical two component system operon. In *F. novicida*, two intact two-component systems are annotated: FTN_1452-FTN_1453 (*bfpR* and its paired sensor kinase *bfpK*) and FTN_1714-FTN_1715 (*kdpED*). In addition, *F. novicida* has one orphan sensor kinase FTN_1617 (*qseC*), and one orphan response regulator *qseB/pmrA* (FTN_1465). The sole response regulator in *F. tularensis* LVS is FTL_0552 (*qseB/pmrA*) with one orphan sensor kinase, FTL_1762 (*qseC*). Alignment of *kdpD* with the LVS genome reveals two genes that align with the first and second half of *kdpD* (FLT_1878-1879), but whose function is not yet defined. *BfpR* is conserved in all the highly virulent *Francisella* strains as well as in *F. novicida* but is highly truncated in the live vaccine strain.

Only three response regulator (RR) genes have been identified in the *Francisella* genus. The two-component system gene organization varies across the different species (Figure 1; Larsson et al., 2005; van Hoek et al., 2019). *F. novicida* has three sensor kinase (SK) genes and three response regulator genes. Two sets of genes form complete two-component system pairs (Figure 1), while the last two genes are “orphaned” (van Hoek et al., 2019). *F. tularensis* Schu S4, the highly infectious human-virulent Type A strain, encodes two sensor kinase genes, two response regulator genes, and two pseudogenes. None of the two-component system genes are intact in the virulent strain *F. tularensis* (Figure 1). Pseudogene sequences contain early stop codons or shifts in the sequence, resulting in a truncated or non-functional gene product. *F. holarctica* Live Vaccine Strain (LVS) has one intact sensor kinase gene (*qseC*), only one response regulator gene, and pseudogenes present in place of the other three genes. Like *F. tularensis*, none of the LVS genes are within intact two-component systems. Finally, in *F. philomiragia*, an environmental strain, and *F. noatunensis*, a fish pathogenic strain, three sensor kinase genes and three response regulator genes were identified, similar to *F. novicida* (van Hoek et al., 2019). We previously investigated the role of the response regulator QseB/PmrA (FTN_1465) in biofilm formation in *F. novicida* (Durham-Colleran et al., 2010; van Hoek, 2013), and demonstrated that biofilm formation is dependent on both the orphan response regulator (QseB/PrmA) and the orphan sensor kinase (QseC, FTN_1617). QseB/PmrA in *F. novicida* is critical for infection and has been identified as a potential virulence factor (Mohapatra et al., 2007; Bell et al., 2010). Overall, the role of the two-component system genes in *Francisella* physiology have not yet been fully elucidated (van Hoek et al., 2019). In this work, we examine the previously uncharacterized *F. novicida* response regulator FTN_1452.

Here, we report our efforts to characterize the *F. novicida* response regulator FTN_1452, which we have named BfpR (Biofilm-regulating *F**rancisella* protein Regulator). We explore the homology of BfpR across *Francisella* species, present the crystal structure of the REC domain, and investigate the impact of BfpR on gene expression and phenotype. Our findings led us to identify a role of BfpR within *F. novicida* including resistance to AMPs and biofilm formation.

## Results and Discussion

### Initial Identification of BfpR in *Francisella*

We began our investigation exploring the conservation of FTN_1452 gene sequences between *Francisella* species and strains. We noted that *F. novicida* FTN_1452 is 100% identical to *F. tularensis* SchuS4 FTT_1543 and >90% identical to the *F. philomiragia* and *F. noatunensis* equivalents (Table 1, Figure S1C). In *F. holarctica* LVS and other *holarctica* strains, there is a truncated version of this response regulator (AJI58249, locus tag AW21_1440). The gene encodes only 123 of the 229 amino acids for the full-length protein. The first 56 and last 57 amino acids are missing, including the conserved aspartate which acts as the phosphoacceptor site (Figure S1E). Since the aspartate is required for response regulator activation, it is predicted that this pseudogene will be inactive.

**Table 1:** Sequence comparison of BfpR with other well-known response regulators.

**Protein**	**Organism**	**E-value**	**Identity (%)**
FTT1543	*Francisella tularensis*	−168	100
MprA	*Stenotrophomonas maltophilia*	−59	41
PhoP	*Xanthomonas campestris*	−49	39
CopR	*Pseudomonas aeruginosa*	−51	38
WalR	*Staphylococcus aureus*	−43	38
MprA	*Mycobacterium tuberculosis*	−54	37
PhoP	*Listeria monocytogenes*	−46	37
YedW	*Salmonella typhimurium*	−46	37
PhoP	*Bacillus subtilis*	−46	37
PhoP	*Staphylococcus epidermidis*	−44	35
CopR	*Escherichia coli*	−47	34
IrlR	*Burkholderia pseudomallei*	−40	33

*Sequence homology comparison of BfpR protein of F. novicida and response regulators of other selected bacterial pathogens. Only the most highly homologous protein from each organism is shown. This sequence comparison suggests that BfpR is not highly homologous by sequence to any known response regulators of other bacterial pathogens*.

Alignments of FTN_452 to other bacterial response regulators (Table 1, Figure S1A) suggest that FTN_1452 is a member of the OmpR/PhoP family (Figure S1D). While FTN_1452 shares sequence similarity around the phosphorylation activity site, overall homology is only significant within the genus *Francisella* (Figures S1A–C). Sequence analysis showed low levels of similarity by sequence to WalR of *Staphylococcus aureus* (38%), PhoP of *Pseudomonas aeruginosa* (40%), and MprA of *Mycobacterium tuberculosis* (37%) (Table 1). Thus, FTN_1452 could not be named as a close homolog to any known response regulator in other bacteria and was given the name BfpR. BfpR stands for Biofilm-regulating *Francisella* protein Response regulator, for its involvement in biofilm formation as a significant phenotype (see below). NCBI conserved domain analysis (Figure S1D) also confirmed that BfpR is composed of a YesN/AraC superfamily REC domain and a helix-turn-helix DNA-binding domain. These conserved domains are highly characteristic of bacterial two-component system response regulators. Using BLAST analysis of *Francisella* genomic sequences, we further confirmed that BfpR in *F. novicida* is identical to FTT_1543 in *F. tularensis* Schu S4, and this gene does not have any close homologs outside of the genus *Francisella* (Figure S1). Thus, by this analysis, BfpR is likely to be a response regulator involved in bacterial signal transduction mechanisms and regulation of transcription in *F. novicida*.

In *F. novicida, bfpR* is encoded in a presumed operon with a sensor kinase, FTN_1453, making a complete two-component system with the response regulator gene appearing first and the sensor kinase gene second in the proposed operon. Interestingly, the sensor kinase gene is absent in *F. tularensis* SchuS4, but is present in the environmental and piscine strains, such as *F. philomiragia* and *F. noatunensis* (van Hoek et al., 2019). The absence of a cognate sensor kinase in the human-virulent strain of *F. tularensis* (FTT_1542 is a pseudogene) suggests that *bfpR* may have to act as an orphaned TCS gene in the virulent *F. tularensis*. Alternatively, perhaps the fact that *F. tularensis* has a duplicated *Francisella* pathogenicity island (FPI) compared to *F. novicida* reduces the need for this TCS in the virulent strain.

Two-component systems can regulate environmental survival as well as virulence of bacteria, among other pathways. No published virulence factor screening studies have identified the FTT_1542 or FTN_1452 gene as required for *Francisella* infection in various models (Moule et al., 2010). This is consistent with the results shown below in which the transposon insertion mutant of *bfpR* shows little or a positive effect on intracellular replication, while overexpression of *bfpR* leads to significant inhibition in intracellular replication. Additional experiments detailing the effect of the *bfpR* mutant and its ability to interact with potential signaling partners would be needed to fully understand this outcome. Since *F. novicida* is considered more of an environmental organism, while *F. tularensis* is a eukaryotic pathogen, the role of the BfpR response regulator may be more significant when measured with respect to phenotypes that may promote environmental survival, such as biofilm formation.

### Structure of BfpR N-Terminal Receiver Domain

**Figure 2:**
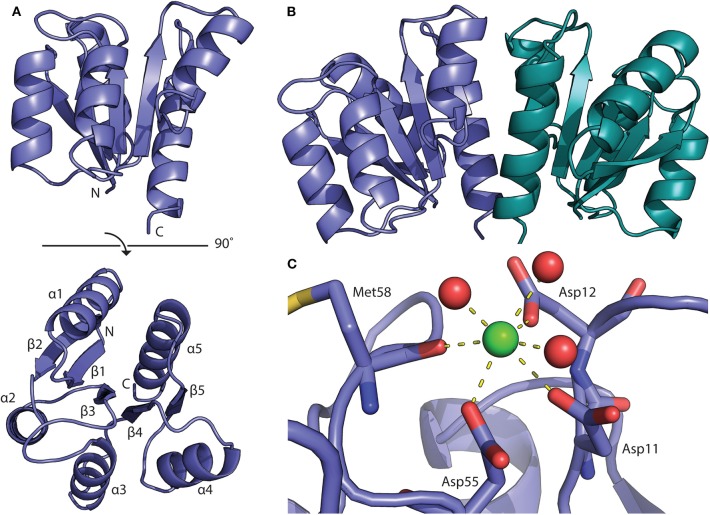
Structure of the BfpR receiver domain. **(A)** Crystal structure of the N-terminal BfpR receiver domain solved to 1.8 Å. **(B)** Structure of BfpR receiver domain dimer with one monomer in blue and one in green. An adjacent symmetry mate forms the biologically relevant dimer. **(C)** Phosphorylation occurs at the conserved aspartate residue (Asp55). A calcium ion (green spheres) is located in the active site and is coordinated with active site residues and water molecules (red spheres) as seen in crystal structure of other phosphorylated response regulators.

The structure of the BfpR N-terminal REC domain (BfpRN) was solved using X-ray crystallography (Figure 2A). Cubic crystals grew within 48 h in 0.2 M calcium chloride, 0.1 M HEPES pH 7.5, and 28% polyethylene glycol 400 using hanging-drop vapor diffusion. The resulting crystals had an I4_1_32 space group and unit cell dimensions of a = b = c = 128.726 Å and α = β = γ = 90° (Table S2). The N-terminal domain of QseB from *F. novicida* [PDB ID 5UIC, (Milton et al., 2017)] was used as the model for the molecular replacement solution. The structure was refined to 1.80 Å resolution with a crystallographic *R*_*work*_ of 0.1824 and *R*_*free*_ of 0.2019. Refinement resulted in no Ramachandran outliers and 98.32% favored. The BfpRN construct used for crystallization was composed of residues Met1 to Ala129 with three additional N-terminal residues remaining after cleavage of the affinity tag. Only residues Asn4 through Lys124 could be traced into the electron density.

**Figure 3:**
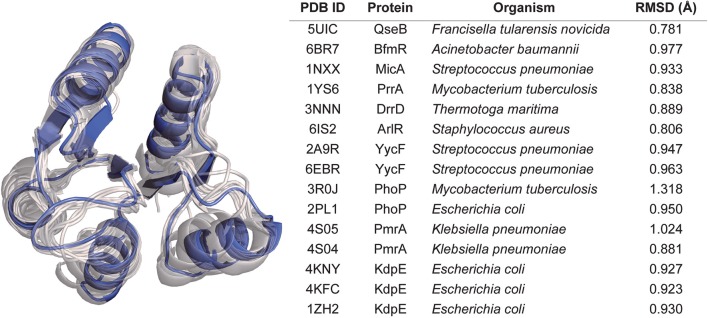
Structural comparison of BfpR and other response regulators. Alignment of our BfpR structure to 15 other response regulators in the Protein Data Bank (PDB) demonstrates the high level of structural similarity between the BfpR and other OmpR/PhoP response regulator receiver domains. BfpR is shown in blue (left) with other response regulators in gray. The table (right) denotes the PDB ID for each structure used along with the Cα RMSD. RMSD values were calculated from the alignment of each PDB entry to BfpR in PyMol.

As expected from our sequence homology observations, the structure shares the canonical OmpR/PhoP receiver domain with an alternating α/β-fold consisting of 5-helices and 5-strands (Figure 3; Bourret, 2010). Structural alignment of BfpR with an array of other OmpR/PhoP family receiver domains the high level of structural similarity between the receive domain of these response regulators. On average, the Cα backbone align with an average RMSD of about 0.9 Å across ~110 atoms (Figure 3). The asymmetric unit of BfpR contains one monomer with an adjacent symmetry mate forming the biologically relevant dimer (Figure 2B) as described previously (Milton et al., 2017; Draughn et al., 2018). The dimer α4-β5-α5 interface of BfpR contains major salt bridges between Lys92 and Glu112, Asp102 and Arg116, and Asp101 and Arg123. These residues have been shown to have homology in other OmpR/PhoP family response regulators (Draughn et al., 2018). The BfpR active site contains the canonical conserved aspartate residues, including the phosphorylation site at Asp55. A calcium ion is coordinated into the position of the active site magnesium (Figure 2C). It has been previously demonstrated that the crystal lattice appears to trap the response regulator in an active state, even in the absence of phosphate or a phosphomimic (Draughn et al., 2018). This was demonstrated with the response regulator BfmR, the master biofilm regulator from *Acinetobacter baumannii*. Alignment of active site residues of BfpR to BfmR bound to a phosphomimic results in an RMS of 0.290 Å. This strongly suggests that BfpR contains a functional phosphorylation site.

### Transcriptomic Analysis of *bfpR* Mutant and *bfpR* Overexpressing Strain

To investigate the effect of BfpR on gene transcription in *F. novicida*, we performed RNAseq analysis. We assessed the effect of the *bfpR* transposon-insertion mutant (Gallagher et al., 2007) and by overexpressing *bfpR* using the *Francisella gro* promoter. The overexpression of response regulators and other transcriptional regulators has been previously found to be a useful method for understanding the impact of a specific regulator on *Escherichia coli* and *Saccharomyces cerevisiae* (Nishino and Yamaguchi, 2001; Tanaka et al., 2012). We used this method for studying BfpR's effect on gene expression and other outputs throughout this study in *F. novicida*.

**Figure 4:**
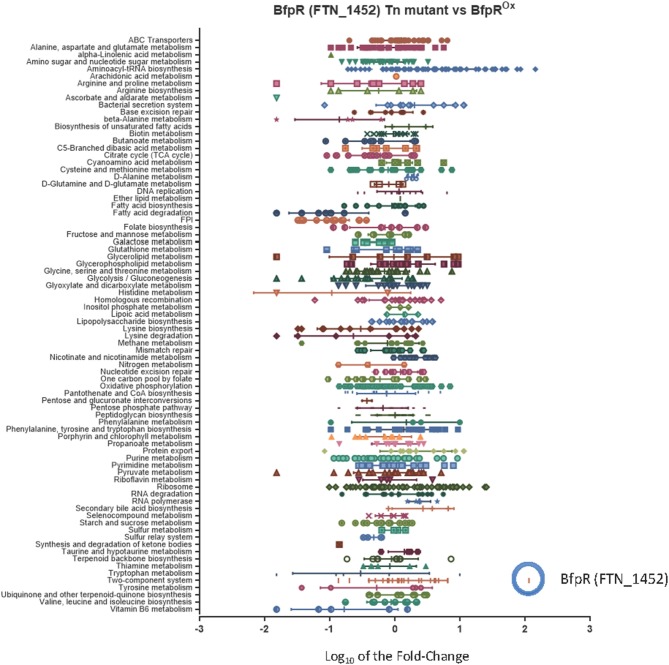
Characterization of the *bfpR* regulon. The categorization of the *bfpR* regulon by mapping the RNASeq fold-change data to KEGG Pathways, with the addition of the *Francisella* Pathogenicity Island (FPI). The graph illustrates the RNASeq results for Log_10_ fold-change in expression comparing *bfpR* Tn-mutant (FTN_1452 Tn-mutant) and *bfrR*^*ox*^ mutant. The change in expression of *bfpR* gene in the overexpressor (blue circle) was one of the most significant with a log_10_ fold change for *bfpR* of 2.06 (114 fold change, Table S3, Figure S2B). The FPI gene cluster is indicated with the blue arrow.

We compared wild type *F. novicida* (WT) gene expression to a *bfpR* transposon-insertion mutant (Gallagher et al., 2007) and the BfpR overexpressing strain (*bfpR*^*ox*^) (Table S1). While RNA-Seq data is typically presented as the expression level in the *bfpR* mutant compared to the WT, we also calculated the ratio change in the *bfpR*^*ox*^ overexpression mutant vs. the *bfpR* mutant (Figure S2A). The results confirmed the high level of *bfpR*^*ox*^ compared to WT (Figure S2B). The results indicate broad effects of *bfpR* and the *bfpR*^*ox*^ on *F. novicida*, including a down-regulation of fatty acid metabolism and the FPI genes in both the *bfpR*^*ox*^ vs*. bfpR* and WT vs. *bfpR* comparisons (Figure 4).

Many proteins encoded by the *Francisella* Pathogenicity Island (FPI) have been shown to be virulence factors, especially intracellular growth locus C, *iglC* (Nano et al., 2004). As part of its intracellular replication, *F. tularensis* escapes from the phagosome into the cytoplasm in an *iglC-*dependent manner where it replicates further, making iglC a required virulence factor for *Francisella* (Lai et al., 2004; Lindgren et al., 2004). *IglC* is part of the newly discovered Type VI secretion system in *Francisella*. Using KEGG analysis of the RNAseq data, the entire FPI was a significantly down-regulated pathway (down an average of 5-fold), with intracellular growth locus A (*iglA*) down-regulated ~18-fold, *iglC* expression downregulated ~15 fold, and all members of the FPI significantly changed in the *bfpR*^*ox*^ (Figure 4, Figure S2A). This suggests that the pathogenicity of the *bfpR*^*ox*^ bacteria may be significantly reduced, as iglC expression (downregulated ~15 fold in the *bfpR*^*ox*^) is required for intracellular replication and pathogenesis (Lai et al., 2004). As an example of the effect on metabolic genes, alcohol dehydrogenase, *adhC*, was down 32-fold, and members of the *fad* operon significantly changed in the both mutant and *bfpR*^*ox*^ comparisons.

Gene expression of the acid phosphatase AcpA was not significantly changed by overexpression of *bfpR* but changed (~2-fold) in the WT vs. *bfpR* comparison. Importantly, the *bfpR*^*ox*^ strain displayed a >100-fold increase in *bfpR* transcripts compared to WT and *bfpR* mutant, confirming that this strain was indeed overexpressing *bfpR* (Figures S2A,B). Of all the TCS genes, *qseB* (FTN_1465) was the least affected by *bfpR*^*ox*^ (Figure S2B). Overall, these results suggest the broad range of genes controlled directly or indirectly by BfpR.

In comparing the proteomic (Figure S2C) and transcriptomic (Figure S2A) results, a glycogen synthesis gene *glgC* was found to be downregulated (6.5-fold) in *bfpR*^*ox*^ *vs. bfpR*, consistent with its protein expression levels. Proteins of peptidoglycan synthesis as well as Phe, Tyr and Trp biosynthesis proteins were also highly upregulated in *bfpR*^*ox*^, as well as cell division-associated proteins. Other comparisons between the proteomic and transcriptomic results show that in addition to the FPI genes and proteins *iglA, iglB* and *iglC* being strongly down-regulated in *bfpR*^*ox*^, many tRNA genes (e.g., FTN_1541, tRNA Arg) are upregulated in in *bfpR*^*ox*^, corresponding to the increased expression of Arg, Pro metabolism proteins in the proteomic data.

### Effect of *bfpR* Mutation and Overexpression on Protein Expression

We next characterized the BfpR “regulon” using proteomic analysis. This method has been previously used to help define the regulon of PhoP in different strains of *Salmonella enterica* (Charles et al., 2009). The protein expression data shows 342 proteins were dysregulated ≥1.5-fold when comparing *bfpR* and WT, and 413 proteins were up- or down-regulated ≥1.5-fold in the *bfpR*^*ox*^ strain to *bfpR* comparison. One hundred seventy-nine proteins were found to be overlapping between these two comparisons. To further analyze the overlapping region of this dataset, we categorized the proteins by KEGG pathway. Notably, comparing the mutation of *bfpR* and the complementation back caused a significant change in abundance of FPI proteins, with an average percent decrease of 60% in the WT vs. *bfpR* to *bfpR*^*ox*^ vs. *bfpR* comparisons (Figure S2C). Among the many *F. novicida* proteins that changed by proteomics analysis, *IglC* was downregulated by *bfpR*^*ox*^, confirmed in the Western Blot data shown below. Acid phosphatase A, AcpA, was reduced in *bfpR* (2-fold) compared to WT, correlating to the RNASeq data. The level of glucose-1-phosphate adenylyltransferase protein (involved in glycogen synthesis, GlgC) was increased (3-fold) in *bfpR* compared to WT. Thus, unlike the deletion of QseB/PmrA, which positively regulates FPI gene expression and deletion leads to decreased expression of these virulence factors, deletion of BfpR has little direct effect on protein expression. This suggests that BfpR may be a negative regulator unlike QseB/PmrA. Some proteins (such as GlgC see below) appear to be positively regulated by *bfpR* mutation, illustrating the potential complexity of the *bfpR* regulon.

### Putative Target Binding Sequence of BfpR

We next analyzed the promoters of genes found in the *bfpR* regulon, choosing WalR as the model response regulator and the *F. novicida* U112 genome in the program PePPER (de Jong et al., 2012). This led us to identify a putative predicted palindromic binding sequence for BfpR as: TGT-n_8/9_-TGT, which was identified upstream of multiple BfpR-regulated *Francisella* genes, including *iglA, glgC*, and *acpA* (Figures S3B, S4). Formal definition of the BfpR binding site will require DNA footprinting assays and EMSA assays, but our computational prediction of a putative binding palindrome allowed us to explore some aspects of potential BfpR-DNA binding.

To test the binding of BfpR to DNA, we established DNA-protein interaction using ELISA and ChIP-PCR with fragments of the *acpA* promoter. Creating three ~150 bp fragments from −500 to −10 upstream of the start sites, we found through ELISA studies that BfpR-His_6_ binds to the distal region of this promoter (Figure S4A). These results were further indicated by amplification using primers for fragment I in ChiP-PCR experiments (Figure S4B). With both techniques, binding of BfpR-His_6_ to other *acpA* promoter regions was not detected. Circular dichroism experiments of BfpR-His_6_ in the presence and absence of *acpA* promoter fragments further suggested a specific interaction may occur between BfpR and the distal region of the promoter sequence (Figure S4C). These results are consistent with the predicted TGT-n_8/9_-TGT binding site, found in the distal fragment.

To further evaluate DNA binding, we explored *in silico* docking of a small DNA fragment to a full-length homology model of BfpR (Figure S3A). HADDOCK (Wassenaar et al., 2012; van Zundert et al., 2016) was used to probe the binding interactions between an *acpA* promoter fragment 5′-AACTGTTAC and our full-length model of BfpR. The model was generated by combining the crystal structure of the N-terminal domain with other structures of full-length OmpR/PhoP family response regulators. HADDOCK clustered 167 structures into 12 clusters. The top scoring cluster based on the HADDOCK score and Z-score clearly places the DNA fragment onto the canonical DNA-binding helix of the BfpR DNA-binding domain (Figure S3B). This cluster has significantly better scores than the next best ranked cluster, indicating that this DNA binding position is the best docking solution. These results suggest that BfpR should bind DNA in a similar manner as other OmpR/PhoP response regulators and suggests a putative palindromic sequence.

To confirm the binding of BfpR to a shorter, more specific sequence we generated small DNA fragments containing TGT-n_8/9_-TGT from *acpA, iglA, pgm*, and a mutated *acpA* fragment where TGT was replaced by CAC (Figure S4D). DNA-protein interaction ELISA showed that *acpA, iglA*, and *pgm* fragments bind (*p* < 0.01) to BfpR-His_6_, but not the mutated CAC-n_8/9_-CAC fragment (Figure S4E). With the binding sequence better established, we aligned the TGT-n_8/9_-TGT segments of genes found within the BfpR regulon (the set of genes regulated by BfpR) identified above to generate the putative consensus sequence: TGT(T/A)X(T/A)XXX(T/A)XXTGT (Figure S4F) as the putative predicted BfpR binding sequence.

### BfpR Is Associated With Regulation of Biofilm Formation

**Figure 5:**
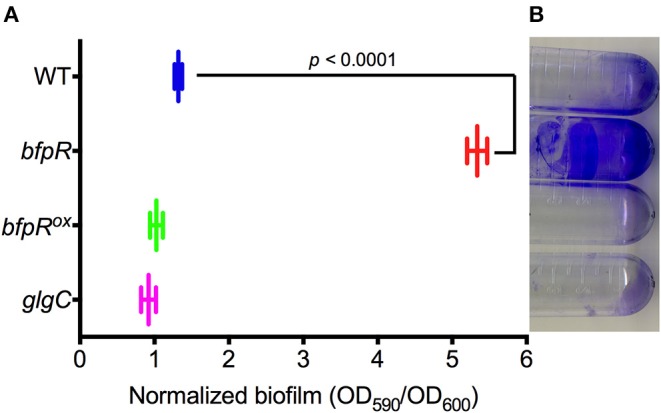
Impact of *bfpR* on biofilm formation. **(A)** Quantification of biofilm formation of WT, *bfpR, bfpR*^*ox*^, and *glgC*, measuring crystal violet staining from triplicate experiments. *bfpR* produced significantly more (*p* < 0.0001) biofilm than WT, *bfpR*^*ox*^, and *glgC*. **(B)** Representative image of biofilm formation on polystyrene tubes.

Response regulators are known to regulate biofilm formation and dispersal in other bacterial systems. Our group has previously showed the positive regulation of biofilm formation by the QseB/PmrA response regulator in *F. novicida* (Durham-Colleran et al., 2010). Mutation or deletion of QseB/PmrA leads to a significant decrease in biofilm formation. Here, we assessed the role of BfpR in biofilm formation in *F. novicida* by measuring the ability of WT, *bfpR*, and *bfpR*^*ox*^ to form biofilm. Using the crystal violet staining method, biofilm adherence to polystyrene was quantified as in our previous study (Durham-Colleran et al., 2010). The *bfpR* mutant had a ~5-fold increase in biofilm formation over WT (Figure 5). The results also indicate that WT and *bfpR*^*ox*^ produced statistically equivalent amounts of biofilm after 24 h, which were both much less than the large amount of biofilm produced by the *bfpR* mutant. The biofilm production of the *glgC* transposon-insertion mutant is also demonstrated. The *bfpR* mutant phenotype is the opposite phenotype than the QseB/PmrA mutation, which showed a decrease in biofilm production.

Because BfpR protein is predicted to bind to *pgm-glgC* promoter DNA (Figure S4F), we tested whether *bfpR* mutation influenced the levels of intracellular glycogen and extracellular polysaccharide (*pgm* is immediately upstream of *glgC* and the two genes are predicted to be in the same operon, sharing the same promoter). We demonstrated that GlgC protein levels were 3-fold increased in the *bfpR* mutant strain (see above). The extracellular level of polysaccharide production from WT, *bfpR*, and *bfpR*^*ox*^ was determined by iodine vapor staining. The *glgC* transposon-insertion mutant (Gallagher et al., 2007) was used as a control for no glycogen production. Figure S6A shows that *bfpR* mutation increases the level of polysaccharide formed, while WT and *bfpR*^*ox*^ have lower levels. The staining using iodine is not specific for any one polysaccharide and other macromolecules can complex with iodine, but one of the polysaccharides stained by iodine is glycogen. Next, we quantified the intracellular glycogen levels in WT, *bfpR*, and *bfpR*^*ox*^ using a colorimetric glycogen assay. The *glgC* mutant was used as a control for no glycogen production. Figure S6B shows the relative higher glycogen levels in the *bfpR* mutant, compared to WT and *bfpR*^*ox*^. This increased polysaccharide formation could be contributing to the significant increase in biofilm production we observed.

Thus, the *bfpR* mutant makes more biofilm, more general polysaccharide and more glycogen than wild-type *F. novicida*. The expression of glycogen synthesis genes *glgX* (FTN_0512) and *glgB*-*pgm-glgCAP* (FTN_0513-FTN_0517) are altered in response to *bfpR* overexpression (1.46-fold for glgX; 0.42, 0.377, 0.145, 0.565, 0.436-fold, respectively, for the rest), while *pgm* (FTN_0514) and *glgP* (FTN_0517) were down regulated in the *bfpR* mutant vs. WT (0.466-fold and 0.551-fold, respectively). Our results indicated that mutation of *bfpR* increased biofilm formation and polysaccharide levels, as well as intracellular glycogen, while in *bfpR*^*ox*^ these levels were significantly decreased. The regulation of the differently organized *glgBXCAP* operon in *E. coli* by the PhoP-PhoQ TCS provides evidence of similar regulation in another Gram-negative bacteria (Montero et al., 2011). Both *glgB* and *glgC* have been shown to be essential for *Francisella* lung infection in mice (Su et al., 2007), and mutants of *glgB* were shown to be attenuated in a *Drosophila melanogaster* infection model (Ahlund et al., 2010). The connection between the glycogen synthesis genes with biofilm formation and virulence has been established in other Gram-negative organisms (McMeechan et al., 2005; Ito et al., 2009); however, the connection between glycogen synthesis genes, biofilm formation and virulence in *Francisella* remains to be further characterized.

### *bfpR* Mutation Increases Susceptibility to AMPs and Overexpression Increases AMP Resistance

Other phenotypes of interest in *Francisella* include cationic antimicrobial peptide (AMP) resistance (Jones et al., 2012; Li et al., 2012) and intracellular growth (Nano et al., 2004; Jones et al., 2012), each of which has been demonstrated as being under the control of response regulator in other bacteria. Response regulators are known to control resistance to polymyxin B (cyclic peptide antibiotic) and AMPs, including the response regulators ParR (Fernandez et al., 2010), PhoP (Guina et al., 2000), and PmrA (Gunn and Miller, 1996) in other bacteria. A gene encoding glycosyltransferase (FTN_0545, FlmF2, or yfdH) was found to be significantly deceased (0.45-fold) in expression in the *bfpR* mutant strain relative to WT (Richards et al., 2008; Wang et al., 2009). This gene is involved in the “biosynthesis of disaccharides, oligosaccharides and polysaccharides” and has sequence homology to ArnC of *P. aeruginosa*, which is annotated as a “putative polymyxin resistance protein” (Song et al., 2009). In *P. aeruginosa*, the *arn* operon is involved in resistance to peptide antibiotics and cathelicidin AMPs such as the human LL-37, and is regulated by the TCS ParR-ParS (Fernandez et al., 2010). The altered expression of LPS-related genes (Figure 4) in the *bfpR* mutant and *bfpR* complemented overexpressing strain (*bfpR*^*ox*^) is also consistent with the respective increased susceptibility and resistance to cationic AMPs in these two strains.

**Figure 6:**
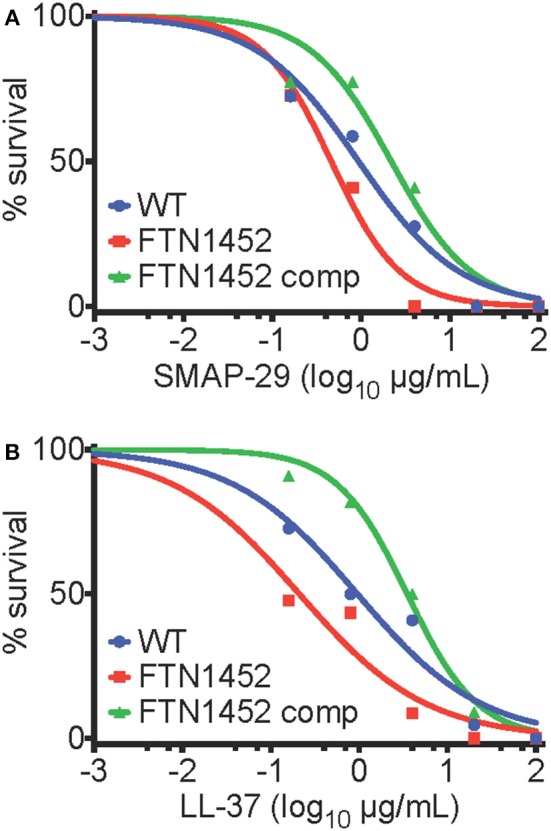
*bfpR* influences AMP resistance. **(A,B)** Survival of bacteria with titration of cathelicidin AMPs. **(A)** EC_50_ values of WT, *bfpR*, and *bfpR*^*ox*^ with LL-37 were found to be 1.0, 0.2, and 3.5 μg/mL, respectively. **(B)** EC_50_ values of WT, *bfpR*, and *bfpR*^*ox*^ with SMAP-29 were found to be 0.9, 0.5, and 2.2 μg/mL, respectively. Data are shown are a representative of three independent experiments.

The effect of AMPs and peptide antibiotics on WT *F. novicida* and *bfpR* mutants was tested. *bfpR*^*ox*^ was significantly more resistant to AMPs LL-37 and SMAP-29 than WT (*p* < 0.05) (Figure 6). Specifically, the EC_50_ values (and associated 95 % confidence intervals) of WT, *bfpR*, and *bfpR*^*ox*^ for LL-37 were determined to be 1.0 (0.4–2.4), 0.2 (0.1–0.8), and 3.5 (2.5–5.0) μg/mL, respectively, for SMAP-29 the values were 0.9 (0.5–1.8), 0.5 (0.3–0.7), and 2.2 (0.9–5.3) μg/mL, respectively. The EC_50_ values obtained show significant ≥2-fold shifts in sensitivity and resistance in a BfpR-dependent manner. Our study showed slightly increased susceptibility of *F. novicida* to two potent alpha-helical AMPs (LL-37 and SMAP-29) with *bfpR* mutation and significantly increased resistance when *bfpR* was overexpressed.

*Francisella* species are known to be highly resistant to polymyxin, a drug that is often used as a model of cationic antimicrobial peptide sensitivity. *Francisella* grown on media containing polymyxin B at 100 μg/ml in the specialized media used to isolate *Francisella* spp. from environmental samples (Petersen et al., 2009). *Francisella* that have the mutant *bfpR* were not found to be more susceptible than WT to cyclic peptide antibiotics such as polymyxin B, while *bfpR*^*ox*^ displayed significant resistance to killing (Figure S7). Thus, BfpR may be a positive regulator of AMP-resistance, likely through an indirect mechanism.

To ascertain whether the AMP resistance of *bfpR*^*ox*^ was to antibiotics in general, other compounds were tested. We determined that the susceptibility to streptomycin, ciprofloxacin, and gentamicin was not significantly altered by *bfpR* mutation or overexpression (see Figure S7B).

### High Mg^2+^ and Response Regulator-Targeting Inhibitor Recovers Growth Inhibited by *bfpR* Overexpression

**Figure 7:**
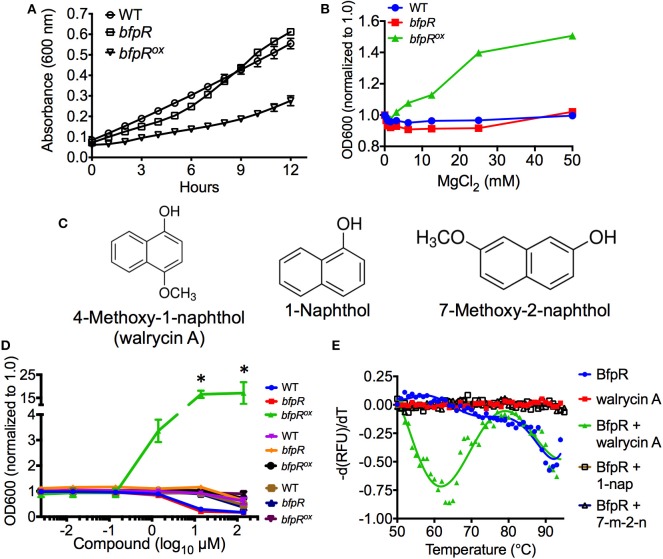
*bfpR* overexpression growth defect and growth induction by Mg^2+^ and inhibitor. **(A)** The growth rates of WT and *bfpR* in TSBC. **(B)** The growth of WT and *bfpR* in TSBC with increasing concentration of MgCl_2_. **(C)** Chemical structures of walrycin A (4-Methoxy-1-naphthol), 1-Naphthol, and 7-Methoxy-2-naphthol are shown. **(D)** WT, *bfpR*, and *bfpR*^*ox*^ with a titration of walrycin A (blue, red, green) and control compounds 1-naphthol (purple, orange, black) and 7-Methoxy-2-naphthol (brown, navy blue, and maroon). The data normalized to 1.0 for untreated. **(E)** Differential scanning fluorimetry using SYPRO Orange was performed on BfpR-His_6_ with and without walrycin A, 1-Naphthol, or 7-Methoxy-2-naphthol. Walrycin A: wal A, 1-Naphthol: 1-nap, 7-ethoxy-2-naphthol: 7-m-2-n. Data are shown as averages from three independent experiments, error bars indicate standard deviation, and significance was determined by Student's *t*-test. *p < 0.001.

Mg^2+^ is often sensed via bacterial two-component systems, such as PhoPQ, where under high Mg^2+^ PhoQ dephosphorylates PhoP to an inactive state (Groisman, 2001). We assessed the growth of the *bfpR* mutant and *bfpR*^*ox*^ and observed a significant inhibition of growth (*p* < 0.05) from overexpressing *bfpR* (Figure 7A). Our results show that Mg^2+^, in high concentrations (100 mM), did not alter the growth of WT or *bfpR* mutant bacteria (Figure 7B). In the growth deficient *bfpR*^*ox*^, however, Mg^2+^ increased growth back to WT levels in a dose dependent manner (Figure S8). Thus, high Mg^2+^ released the growth suppression induced by overexpressing BfpR and relieves the apparent growth defect.

The relationship of the signal Mg^2+^ and gene regulation via the PhoP-PhoQ TCS has been established in *Salmonella typhimurium, E. coli, Yersinia pestis*, and *P. aeruginosa* (Garcia Vescovi et al., 1996; Minagawa et al., 2003; Zhou et al., 2005; McPhee et al., 2006). Although *Francisella species* do not encode for an obviously homologous PhoP-PhoQ system, *bfpR* and its adjacent putative sensor kinase *bfpK* may play a similar role in *F. novicida* signaling. Our growth and kinetic experiments showed that the growth defect of the *bfpR*^*ox*^ strain could be relieved by high Mg^2+^ treatment (similar to the effect of walrycin A, a response regulator-inhibitor, shown below). We have not yet determined whether this Mg^2+^ effect is directly acting on the BfpR or is an indirect action on the cognate sensor kinase.

Interestingly, a similar effect was seen using a response regulator-inhibitor found to target *WalR* in Gram-positive bacteria, walrycin A (Gotoh et al., 2010). We tested walrycin A (4-methoxy-1-naphthol) and two control naphthol compounds (Figure 7C) for their effect on growth of WT, *bfpR*, and *bfpR*^*ox*^. The control compounds, 1-naphthol and 7-methoxy-2-naphthol, differ only slightly from walrycin A in their chemical structure. Walrycin A alone was capable of offsetting the decreased growth of *bfpR*^*ox*^ (*p* < 0.05) (Figure 7D).

To explore potential binding of walrycin A to BfpR, computational docking experiments were performed using AutoDock 4.2. Walrycin A, 1-naphthol, and 7-methoxy-2-naphthol were each docked to our full-length model of BfpR. The location of the top ranked putative binding pocket (pocket 1) was the same for all three compounds (Figure S9). Pockets 2 and 3 also ranked among the top binding sites across all the compounds. Pockets 1 and 2 are located in the DNA binding domain. Pocket 2 is specifically located at the end of the DNA-binding helix where compound binding could directly impact DNA-binding activity. Pocket 3 is located within the dimerization interface of the N-terminal receiver domain where a compound could disrupt the ability of BfpR to dimerize. From the putative binding interactions identified in these docking simulations, the ability of walrycin A to offset the decrease in *bfpR*^*ox*^ growth could be caused by walrycin A binding BfpR at these sites. There is no significant difference between the predicted interactions between BfpR and walrycin A, 1-naphthol, and 7-methoxy-2-naphthol. This is likely due to the similar structural characteristics of each of these compounds. The predicted binding energy, ligand efficiency (LE), and binding efficiency index (BEI) for each compound in each pocket are shown in Figure S9. Based on these results, walrycin A, 1-naphthol, and 7-methoxy-2-naphthol all have the potential to bind BfpR within the limits of rigid docking experiments.

To confirm these potential binding interactions, we performed differential scanning fluorimetry experiments using BfpR-His_6_ with or without walrycin A, 1-naphthol, and 7-methoxy-2-naphthol. The results show that a protein population not seen in the presence of the control compounds appears in the presence of walrycin A (Figure 7E). Consistent with the proposed mechanism on WalR (Gotoh et al., 2010), the thermal shift to lower temperatures suggests that the walrycin A-treated BfpR population is destabilized and unfolds at a lower temperature than untreated BfpR.

Using a 2-aminoimidazole-based compound that targets response regulators, we have previously shown that we can inhibit BfmR, a master controller of biofilm in *Acinetobacter baumannii* (Thompson et al., 2012; Milton et al., 2017), and *F. novicida* QseB (Milton et al., 2017; Draughn et al., 2018). These results suggest that response regulators are a potential therapeutic target. Walrycin A was discovered in a screen by Gotoh et al. to target the WalR response regulator of *B. subtilis* and *S. aureus* (Gotoh et al., 2010). In both of these low-GC content Gram-positives, WalR is known to be essential for cell viability, while also regulating biofilm formation, antibiotic resistance, and virulence (Dubrac et al., 2007; Howden et al., 2011). Gotoh *et al*. determined that walrycin A binds to WalR monomers, inducing the formation of an unstable dimer, altering the structural conformation such that DNA-binding is inhibited (Gotoh et al., 2010). Two of the top predicted walrycin A binding sites also are located in regions that could directly impact DNA-binding and dimerization. The effect on *F. novicida* appeared to be similar to *B. subtilis* and *S. aureus*, as both WT and the *bfpR* mutant had decreased growth in high concentrations of walrycin A. Conversely, when the *bfpR*^*ox*^ was treated with 10 μM walrycin A, a 16-fold increase in growth was observed, suggesting that walrycin A decreases the effect of *BfpR* repression, enabling growth to WT-levels. These data indicate a potential for response regulator-targeting compounds to control *Francisella* growth, and other activities modulated by response regulators. The concept of targeting sensor kinases in *F. tularensis* with an inhibitor has shown potential in a previous study from our group (Rasko et al., 2008).

### *bfpR* Overexpression Inhibits Intramacrophage Replication and Infection in *Galleria mellonella*

**Figure 8:**
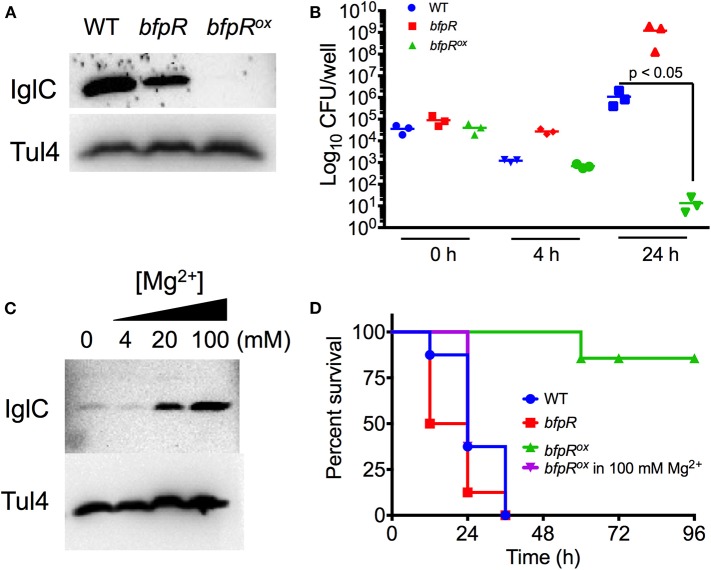
*bfpR* mutation and overexpression affects intracellular replication and virulence. **(A)** Western blot for IglC protein expression in WT, *bfpR*, and *bfpR*^*ox*^. **(B)** Confluent cultures of PMA-differentiated U937 human monocytes were infected (MOI 50). Following the gentamicin protection protocol, CFU was determined. **(C)** Western blot showing IglC expression in the *bfpR*^*ox*^ with increasing concentrations of Mg^2+^. Tul4 was probed as a control protein. **(D)** *Galleria mellonella* were injected with equal CFUs. Caterpillars were observed twice daily for survival status. Data are shown as averages from three independent experiments and significance was determined by Student's *t*-test or log-rank test. The difference between *bfpR*^*ox*^ and the wild type is highly significant, *p* < 0.05.

We assessed the effect of BfpR on intramacrophage replication and virulence in the moth larvae insect model, *Galleria mellonella*. First, to determine the relative levels of FPI protein expression, we assayed levels of one of the well-studied virulence factors, the FPI protein IglC in WT, *bfpR*, and *bfpR*^*ox*^ strains by western blotting. Consistent with our proteomic data, levels of IglC in WT and *bfpR* were similar to each other, while IglC levels in *bfpR*^*ox*^ were significantly decreased (Figure 8A). This response regulator-mediated control of IglC is opposite to the activity shown in previous studies of QseB/PmrA in *F. novicida* and *F. tularensis* LVS (Sammons-Jackson et al., 2008; Bell et al., 2010; Durham-Colleran et al., 2010). In those studies, mutants of *qseB/pmrA* lead to a decrease in IglC expression. Thus, BfpR may act in the opposite direction with respect to *iglC* and FPI gene expression than QseB/PmrA, and be a direct or indirect negative regulator.

Using human-derived U937 macrophages, we infected PMA-differentiated macrophages at 50 MOI for 2 h and performed gentamicin protection so that only intracellular *Francisella* remained. *bfpR* showed increased intracellular levels of bacteria at 4 and 24 h, compared to WT, while *bfpR*^*ox*^ resulted in significantly decreased levels of intracellular bacterial replication after 24 h (*p* < 0.05) (Figure 8B), consistent with the highly reduced iglC expression but this result may also have been affected by the *in vitro* growth defect observed (Figure 7).

To assess the effect of BfpR on *F. novicida* infection, we used the *G. mellonella* infection model. This model organism has previously been shown to be a useful model of the innate immune system in mammals and has been used as a model host of *Francisella* infection (Aperis et al., 2007; Ahmad et al., 2010; McKenney et al., 2012; Kaushal et al., 2016). Figure 8D shows that WT and *bfpR* mutant are similar in pathogenicity to the *Galleria* larvae, while the *bfpR*^*ox*^ is significantly less pathogenic (*p* < 0.001), causing no killing until 60 h, consistent with highly reduced *iglC* expression. We conclude that *bfpR* overexpression reduces virulence of *F. novicida* in this infection model, while *bfpR* transposon insertion mutant has little effect, suggesting a negative regulation model.

Our previous observations indicated that the addition of Mg^2+^ may cause de-repression of the BfpR-mediated decrease in growth. To further investigate this de-repression in relation to virulence factor expression, we incubated *bfpR*^*ox*^ with a titration of Mg^2+^ and probed for IglC levels. Our results show a Mg^2+^ concentration-dependent increase in IglC expression (Figure 8C). To further explore the significance of this result in our infection model, we grew *bfpR*^*ox*^ in 100 mM Mg^2+^ and again, normalized bacterial number and infected *Galleria* larvae. After 36 h the increased virulence of Mg^2+^ *bfpR*^*ox*^ was apparent, with a significantly shorter time-to-death compared to *bfpR*^*ox*^ grown without addition of 100 mM Mg^2+^ (*p* < 0.001), consistent with the de-repression of *iglC* expression. We hypothesize that the initial growth conditions of the bacteria enable the production of certain virulence factors that enable the *Francisella* to establish an infection in the waxworm. Thus, BfpR has sensitivity to Mg^2+^, consistent with its WalR/PhoP classification (Lejona et al., 2003).

The intricate control of the many physiological activities of *Francisella* is critical for its ability to survive in the environment and in infected hosts. The BfpR regulon is shown here to exert physiological effects on *F. novicida*, particularly affecting the production of *F. novicida* biofilm and resistance to antimicrobial peptides. BfpR also indirectly or directly negatively modulates expression of some *Francisella* Pathogenicity Island genes such as *iglC*, which are necessary for intramacrophage replication and infection. In summary, we conclude that *Francisella* BfpR is a response regulator of the OmpR/PhoP family that negatively regulates the expression of some FPI proteins and biofilm formation and positively regulates resistance to AMPs.

Interestingly, this places BfpR in opposition with MglA and QseB/PmrA. The transcriptional regulator MglA and the response regulator QseB/PmrA have been shown to be activators of intracellular replication by positively regulating FPI genes (Brotcke et al., 2006; Dai et al., 2010). However, in a recent study, Ramsey and Dove suggest that QseB/PmrA may not directly regulate FPI expression, and it may not positively regulate expression of FPI genes, therefore the direct role of QseB/PmrA in regulation of virulence remains unclear (Ramsey and Dove, 2016). Further investigation of these two-component systems may allow for a better understanding of the network of proteins controlling virulence factor expression in *F. novicida*, the potential activity of protein complexes that include BfpR, as well as the potential for response regulator-targeting compounds that can control or block the function of these critical proteins.

## Experimental Procedures

### Bacterial Strains, Plasmids, and Growth Conditions

The strains and plasmids used in this study are listed (Table S1). *F. novicida* U112 and *bfpR* transposon mutant (FTN1452, strain tnfn1_pw060328p04q153) obtained from BEI Resources, NIAID, NIH) were grown at 37°C in Tryptic Soy Broth with 0.1% (w/v) cysteine (TSBC). The *bfpR* mutant was grown in kanamycin 20 μg/ml in the first passage to ensure presence of the transposon. *Escherichia coli* was grown in Luria-Bertani (LB) broth or on LB agar. When necessary, antibiotics were added: ampicillin 50 μg/ml, kanamycin 20 μg/ml and tetracycline 50 μg/ml.

### Purifying BfpR N-Terminal Domain

The BfpR N-terminal domain construct (BfpRN, residues 1–129) was cloned into pET28a (Novagen) using NdeI and XhoI restriction sites to generate a protein construct containing a thrombin cleavable N-terminal His_6_ affinity tag. Proteins were over-expressed in BL21(DE3) cells (Invitrogen) at 37°C, 120 rpm in LB. At an OD_600_ of ~0.7, cells were induced with 1 mM isopropyl β-D-thiogalactopyranoside (IPTG) at 16°C, overnight. Harvested cell pellets were stored at −80°C for later use. Pellets were resuspended in lysis buffer (25 mM Tris pH 7.8, 500 mM NaCl, 5 mM imidazole, and 0.1 mM of AEBSF). Cells were sonicated and the resulting lysate clarified at 15,000 rpm for 20 min. The clarified lysate was loaded onto 10 mL of Ni-NTA resin (QIAGEN) pre-equilibrated in lysis buffer. Ni-NTA-bound protein was subsequently washed with 10 column volumes of lysis buffer followed by 10 column volumes of 20 mM Tris pH 7.8, 1 M NaCl, and 5 mM imidazole. The protein was eluted with a linear gradient from lysis buffer to elution buffer (25 mM Tris pH 7.9, 500 mM NaCl, and 300 mM imidazole). Fractions containing protein were pooled. The affinity tag was then cleaved by 100 units of thrombin for 2 h at room temperature. Cleavage was quenched with 0.1 mM AEBSF. The sample was concentrated and further purified using a S100 26/60 size exclusion column (GE Healthcare) equilibrated in 20 mM Tris pH 7.8 and 400 mM NaCl. Fractions containing the purest protein based on SDS-PAGE analysis were pooled and concentrated to 6.7 mg mL^−1^. Protein was stored at 4°C for use in crystallography experiments.

### Crystallization and Structure Determination

BfpRN crystals were grown through hanging-drop vapor diffusion against 0.2 M calcium chloride, 0.1 M HEPES pH 7.5, and 28% polyethylene glycol 400 at room temperature. Crystals grew at 6.7 mg mL^−1^ with a protein to cocktail ratio of 1:1. Crystals were cryoprotected using 30% glycerol prior to flash freezing with liquid nitrogen. Data was collected at a wavelength of 1.0332 Å at APS beamline 23-ID (GM/CA). Diffraction was indexed, merged, and scaled using HKL2000 (Otwinowski and Minor, 1997). Molecular replacement was carried out in Phaser-MR within the PHENIX suite (McCoy et al., 2007; Adams et al., 2010) using our QseBN structure (PDB ID 5UIC) (Milton et al., 2017) as a starting model. The resulting solution was further refined using COOT (Emsley and Cowtan, 2004) and phenix.refine (Adams et al., 2010). Statistics for data collection and refinement are in Table S2. All structure figures were produced using PyMOL (Schrodinger, 2010).

### Construction of Complemented Mutant Overexpressing bfpR

To obtain the *BfpR* gene from WT *F. novicida* U112 cDNA, PCR was performed using forward and reverse primers with the Qiagen HotStarTaq Plus Master Mix Kit (Qiagen). The PCR product was cleaved with PstI and EcoRI (New England Biolabs), run on 1% TAE agarose gel and extracted using the QIAquick Gel Kit (Qiagen). The JSG2845 vector contains a *F. tularensis* LVS *groEL* promoter in front of *pmrA* (Mohapatra et al., 2007). The *pmrA* gene was removed, a multicloning site was added containing PstI and EcoRI, and BfpR was inserted. The plasmid was ligated with T4 DNA ligase (Promega) following manufacturer's instructions. Following ligation, the plasmid was transformed into NEB 5-alpha competent cells (New England Biolabs), and plated on LB agar (10 μg/mL tetracycline). The plasmid was purified using the Qiagen Spin MiniPrep Kit (Qiagen) and sequenced (Macrogen USA, Maryland). Electroporation of the *bfpR* transposon mutant was performed as previously described (Maier et al., 2004). An overnight culture of *bfpR* mutant was pelleted, washed three times in 0.5 M sucrose, and pipetted into the electroporation cell (0.2 cm) and was electroporated (GenePulser II (Bio-Rad, California, US); 600 ohm, 2.5 kV, 25 μF). Electroporated samples were plated (TSBC, 10 μg/mL tetracycline). This method was used for both the untagged BfpR complemented overexpressing (*bfpR*^*ox*^) and the BfpR-His_6_-tagged strains (Table S1).

### Purifying BfpR Protein

The *BfpR* gene was inserted into a protein expression vector for purification. PCR amplification of *bfpR* DNA was done from *F. novicida* U112 cDNA using specific primers in Table 1. The product was inserted into pTrcHis II TOPO (TOPO TA cloning kit, Invitrogen) and transformed into *E. coli* One Shot® TOP10 Chemically Competent cells (Life Technologies, Invitrogen). Positive transformant cells were selected with ampicillin (50 μg/ml) on LB agar and verified by PCR using the Xpress forward primer (Invitrogen). Expression of BfpR in cultures was induced by IPTG (1 mM) for 6–8 h and the His_6_-tagged protein was purified using Ni^2+^-NTA resin (Clontech) and eluted with imidazole. Protein was dialyzed with PBS, lyophilized and frozen until use.

### LC-MS/MS Proteomic Analysis

Whole cell lysates from WT *F. novicida, bfpR*, and *bfpR*^*ox*^ were prepared (Figure S5). Tryptic peptides were analyzed by reverse-phase liquid chromatography with nanospray tandem mass spectrometry using a LTQ linear ion trap mass spectrometer (Thermo Electron Corporation) and fused silica capillary column (100 μm × 10 cm; Polymicro Technologies) with a laser-pulled C-18 tip (5 μm, 200-Å pore size; Michrom Bioresources Inc.). After injection, the column was washed (5 min mobile phase A (0.4% acetic acid), and peptides were eluted using a linear gradient of 0% mobile phase B (0.4% acetic acid and 80% acetonitrile) to 50% mobile phase B (30 min, 0.25 μl/min) and then to 100% mobile phase B (5 min). The LTQ mass spectrometer was operated in a data-dependent mode, where each full mass spectrometric scan was followed by five tandem mass spectrometric scans, in which the five most abundant molecular ions were dynamically selected for collision-induced dissociation using a normalized collision energy of 35%. Tandem mass spectra were searched against the *F. novicida* U112 NCBI database with SEQUEST (Pierson et al., 2011).

### Analysis of Proteomic Data and Promoters

Spectral counting was used to compare relative changes in protein abundance. Average protein expression was analyzed by using the biological significance limit of 1.5-fold up-regulation and 0.66-fold down-regulation from the comparisons of both WT vs. *bfpR* and *bfpR*^*ox*^ vs. *bfpR*. Proteins that were not present in any one of the samples (WT, *bfpR* mutant, or *bfpR*^*ox*^) were excluded from the analysis. For categorization of up and down-regulated proteins, modules of the KEGG database were used, with some additions for clarity (adherence and FPI). Analysis of promoters of genes found in the BfpR regulon were performed using PePPER (de Jong et al., 2012), leading us to identify the putative binding sequence as: TGT-n_8/9_-TGT.

### ELISA

The ELISA method for detection of protein-DNA interactions has been previously described (Brand et al., 2010; Liu et al., 2011; Zhang et al., 2013). The ELISA was performed by using pH 9.4 sodium bicarbonate buffer to coat the surface of an EIA/RIA (96-well) medium binding polystyrene plate (Corning, NY, US) with BfpR-His_6_ at 3 μM or BSA in 100 μL of buffer. Following 3 h incubation at RT, the plate was washed three times. The plate was then blocked with poly(dI-dC) at 10 ng/μL, 1 h. After washing, biotinylated promoter fragments were incubated, 1 h. After 3x washing, rabbit anti-biotin HRP conjugate antibody (Abcam) was added at 1:10,000, 1 h. After washing, 1-Step Turbo TMB solution (Pierce) was added, 15 min. The reaction was stopped and read at 450 nm using a BioTek microplate reader (Vermont, United States). Data is shown with BSA control values subtracted.

### Molecular Modeling of Full Length BfpR

A full-length model of BfpR was generated using MODELER v9.12 (Eswar et al., 2006). The model was constructed using the crystal structure of the N-terminal domain of BfpR and homologs PhoP from *Mycobacterium tuberculosis* (PDB ID 3R0J) (Menon and Wang, 2011), PmrA from *Klebsiella pneumoniae* (PDB IDs 4S04 and 4S05) (Lou et al., 2015), KdpE from *E. coli* (PDB IDs 4KFC and 4KNY) (Narayanan et al., 2014), and the receiver domain of QseB from *F. novicida* (PDB ID 5UIC) (Milton et al., 2017). Five hundred models were generated from the sequence alignment of the above-mentioned protein structures with BfpR. Resulting models were scored based on the normalized DOPE (zDOPE) method (Eswar et al., 2006). The structure with the lowest zDOPE score was subsequently run through MolSoft ICM Full Model Builder to generate a fully refined model (MolSoft LLC). Finally, the model was run through PROCHECK and PSVS to evaluate the quality of the model [https://www.ebi.ac.uk/thornton-srv/software/PROCHECK/ and (Bhattacharya et al., 2007)]. The “best” model had the lowest zDOPE score, highest percentage of favored and allowed Ramachandran regions, and lowest MolProbity clash score.

### BfmR-DNA Binding Simulations

Duplex DNA containing the *acpA* promoter fragment sequence 5′-AACTGTTAC was generated using the 3D-DART webserver (van Dijk and Bonvin, 2009). The full-length homology model of BfpR and DNA were input into the HADDOCK webserver (Wassenaar et al., 2012; van Zundert et al., 2016). K178, E198, R205, T219, and 2 G219 were set as active residues and V199 and R203 were set as passive residues for BfpR based on homology to the DNA bound structure of PmrA (PDB ID 4S04) (Lou et al., 2015). For the DNA, C2 was set as a passive residue and T4, G5, and T6 were set as passive based on hydrogen bonding observed in the PmrA structure. The highest ranked cluster was selected based on criteria established by HADDOCK.

### ChIP-PCR

The BfpR-His_6_ plasmid was transformed into the *bfpR* mutant. The transformant was grown up in 40 mL of TSBC with 10 μg/mL of tetracycline overnight. The bacterial culture was fixed with 1% formaldehyde for 1 h at RT. The cells were then pelleted at 3,000 × g for 10 min and 2 mL of PBS was added to solubilize the pellet. This was then lysed with freeze/thaw repeated three times. The lysate was sonicated three by 10 s, in 1 min intervals on ice. Lysate was incubated with 500 μL of Ni resin at RT for 30 min. The resin was washed and eluted from using imidazole buffers as described in the QIAexpressionist protocol (Qiagen). Using the eluted solution PCR was performed using the (reverse primers are 5′ biotinylated) primers for the promoter of *acpA* (fragments I, II, and III). The ORF of *FTN1340* (*acpP*) was used as a negative control.

### Circular Dichroism

CD spectra were collected using a Jasco J-815 spectropolarimeter with a 0.1 cm path-length cuvette. Spectra were recorded from 220 to 280 nm in 2-nm steps in 10 mM sodium phosphate (pH 7.4, 25°C) on samples of BfpR-His_6_ and the *acpA* promoter regions (−500 to −337, −356 to −177, and −198 to −10 bp). Three scans were taken per sample and averaged. Samples were at a concentration of 50 μg/mL of BfpR-His_6_ and 10 μg/mL of DNA.

### RNAseq

RNA was prepared using the Qiagen RNeasy Mini Kit. RNA samples were sent to Otogenetics (Georgia, US) for sequencing. Sequencing data (fastq) of each of the samples was mapped against the *F. novicida* U112 genome (NC_008601, available at NCBI http://www.ncbi.nlm.nih.gov/genome/?term=NC_008601). Mapped data sets were input into BEDtools to determine the hits counts on each of the regions defined in NC_008601. Hits counts of samples were used as input to edgeR (Robinson et al., 2010) for differential gene expression analysis, where the CPM (count per million), *P*-value, and FDR were calculated using Exact test in edgeR, with biological coefficient of variation (BCV) set to 0.1. A false discovery rate (FDR) <0.05 was considered as significantly different. For the WT sample, 12,547,978 reads were obtained. For the BfpR mutant, 10,523,222 reads were obtained as a combined data set from 2 runs, for the complemented overexpression strain BfpR^*ox*^, 12,516,440 reads were obtained. These resulting data were then subjected to analysis similar to that described in *Analysis of proteomic data and promoters* methods above, with cutoff of [log2FC]>2.

### Differential Scanning Fluorimetry

DSF experiments were carried out as previously described (Niesen et al., 2007). Control heating and fluorescence record was done with CFX96 Realtime and C1000 Thermal Cycler (Bio-Rad, California, US). Five micrometers BfpR-His_6_ was loaded into hard shell 96-well PCR plates with transparent film covers (Bio-Rad California, US), with 2X SYPRO Orange (Sigma, Missouri, US). Heat gradients were carried out to 95°C. Fluorescence was analyzed in the presence or absence of walrycin A (4-methoxy-1-naphthol), 7-methoxy-2-naphtol (Santa Cruz Biotech, California, US), or 1-naphthol (Sigma, Missouri, US). Unfolding at lower temperatures, relative to control samples, indicates destabilization.

### BfpR-Small Molecule Docking Simulations

Walrycin A, 1-naphthol, and 7-methoxy-2-naphthol were docked to our full-length model of BfpR using AutoDock 4.2 (Morris et al., 2009). One hundred poses were searched against the BfpR model and clustered using default AutoDock 4.2 settings for the genetic algorithm, with the maximum number of evaluations set to long (25,000,000 evaluations). Top-ranking clusters were analyzed based on binding energy, ligand efficiency, and binding efficiency index and visualized using PyMol.

### Susceptibility to AMPs

Susceptibility to AMPs was assessed using previous described methods for EC_50_ determination (Han et al., 2008; Amer et al., 2010). All peptides were synthesized by ChinaPeptides, Inc., (Shanghai, China) using Fmoc chemistry. Peptides were provided at >95% purity; structure and purity were confirmed via RP-HPLC and ESI-MS. Briefly, AMPs were serially diluted in 10 mM sodium phosphate (pH 7.2). Bacteria were added to each well and incubated for 3 h at 37°C with 5% CO_2_. Samples were then serially diluted and plated on TSBC agar plates as previously described (Dean et al., 2011). Colonies were counted and EC_50_ values were calculated using GraphPad Prism software (version 6) for Mac OS (GraphPad Software, San Diego, CA).

### Biofilm Formation

Overnight cultures of bacteria were diluted 1:30 into 20 mL of TSBC. 200 μL was added to each well of a 96 well plate (BD Falcon 353072). After 24 h at 37°C, the optical densities (OD) of the wells were taken at 600 nm to normalize for growth, then the liquid was removed by washing with tap water as previously described (O'Toole, 2011). Plates were then incubated at 70°C, 1 h and stained with 0.1% (w/v) crystal violet, 15 min. The stain was removed and previous wash step was repeated. Stain was solubilized out from the biofilm by adding 200 μL of 30% (v/v) acetic acid and absorbance was read at 590 nm with a microplate reader. Observation of biofilm formation in test tubes was performed similarly. 1:30 dilutions of overnight cultures were carried out into 3 mL of TSBC. After growth at 24 h at 37°C, absorbance at 600 nm was taken. Bacteria were removed, tubes were washed, and then stained with 0.1% (w/v) crystal violet. The stain was removed, tubes were washed, and pictures were taken with a Perfection 2,480 PHOTO scanner (Epson).

### Western Blots

To determine IglC protein expression, WT, *bfpR*, and *bfpR*^*ox*^ were grown overnight in TSBC, bacterial number normalized by OD600 and pellets lysed with BPER (Pierce). Protein concentration was determined by BCA assay. Samples were run on 4–20% SDS-PAGE and transferred to nitrocellulose, probed with mouse monoclonal antibodies to IglC (NR-3196) and Tul4 (NR-29019, BEI Resources, Manassas, VA) followed by HRP-conjugated goat anti-mouse secondary antibody and developed by SuperSignal West Femto Chemiluminescent Substrate (Pierce). To determine Mg^2+^-dependent IglC protein expression in *bfpR*^*ox*^, the same protocol was followed but with addition varying MgCl_2_ concentrations during growth.

### Intracellular Replication Assays

The U937 cell line (CRL-1593.2), derived from human macrophages, was used to determine intracellular replication activity. Cells were cultivated in RPMI 1640 medium (Thermo) supplemented with 10% heat-inactivated fetal bovine serum (Gibco). One percentage of penicillin-streptomycin (Gibco) was added during initial cultivation. 1 × 10^7^ cells in 30 mL RPMI in 75 cm^2^ flasks were incubated at 37°C with 5% CO_2_. Experiments were not performed on cells passaged more than 30 times. For infections, cells (1 × 10^6^ cells/well in 24 well plates) were differentiated for 24 h using phorbol 12-myristate 13-acetate (PMA) (Sigma, Missouri, US). The differentiated cells were washed with PBS and fresh media was added. Bacteria were added at 50 MOI for 2 h by spinfection. Cells were washed with PBS and fresh media was added with 20 μg/mL gentamicin (Fisher Chemical) and incubated for 1 h. The cells were then washed again and media was replaced with RMPI containing 2 μg/mL gentamicin. Intracellular replication kinetics were determined by lysing with 0.1% deoxycholic acid (Sigma, Missouri, US) in PBS and plating on TSBC agar plates. The assays were performed in triplicate (3 wells per strain) and repeated three independent times.

### *Galleria mellonella* Infection Model

*Galleria mellonella* larvae were obtained from Vanderhorst Wholesale (Saint Marys, OH, USA). Eight to twelve caterpillars of equal size/weight were randomly assigned to each group. Prior to injection, overnight bacterial cultures were normalized to an OD600 of 0.1. Cultures were also plated to confirm viability. A 1 mL tuberculin syringe was used to inject 10[[Inline Image]]μL of bacteria into the hemocoel of each caterpillar. For Mg^2+^ injection, 10 μL of 1M MgCl_2_ (Sigma, Missouri, US) was injected. Control groups included injection with PBS and MgCl_2_ only. The insects were then observed daily for their survival status. Three independent experiments were performed.

### Statistical Analysis

All statistical tests were performed using GraphPad Prism software (version 6) for Mac OS (GraphPad Software, San Diego, CA). A *P* < 0.05 was considered to be significant. In some cases, data are included with a 95% confidence interval, which is a similar level of significance. The Kaplan-Meier curve and log-rank test was used to graph and analyze the caterpillar infection experiments. The Student's *t*-test was used in each other case.

## Data Availability Statement

This article contains previously unpublished data. The structure of BfpRN has been deposited to the Protein Data Bank with PDB entry ID 6ONT.

## Author Contributions

MH and SD conceived the original study. SD performed experiments. JC and MM designed and performed the experiments to solve the RR structure and the RR modeling experiments. All authors contributed to the writing of the manuscript.

### Conflict of Interest

The authors declare that the research was conducted in the absence of any commercial or financial relationships that could be construed as a potential conflict of interest.
