# Supplementary material for: Francisella novicida Two-Component System Response Regulator BfpR Modulates iglC Gene Expression, Antimicrobial Peptide Resistance, and Biofilm Production
Source: Front Cell Infect Microbiol. 2020 Mar 13;10:82. doi: 10.3389/fcimb.2020.00082 (PMC7082314; doi:10.3389/fcimb.2020.00082)
Supplement: Supplementary file 1 [file Data_Sheet_1.pdf]

Supplemental material for:

Francisella novicida **two-component system response regulator BfpR modulates iglC gene expression, antimicrobial peptide resistance, and biofilm production.**

Scott N. Dean,[1] Morgan E. Milton[2], John Cavanagh[2], Monique L. van Hoek[1*]

[1] National Center for Biodefense and Infectious Diseases, and School of Systems Biology, George Mason University, Manassas, Virginia 20110, USA

[2] Department of Biochemistry and Molecular Biology, The Brody School of Medicine, East Carolina University, Greenville, North Carolina 27834, USA

*For correspondence: mvanhoek@gmu.edu

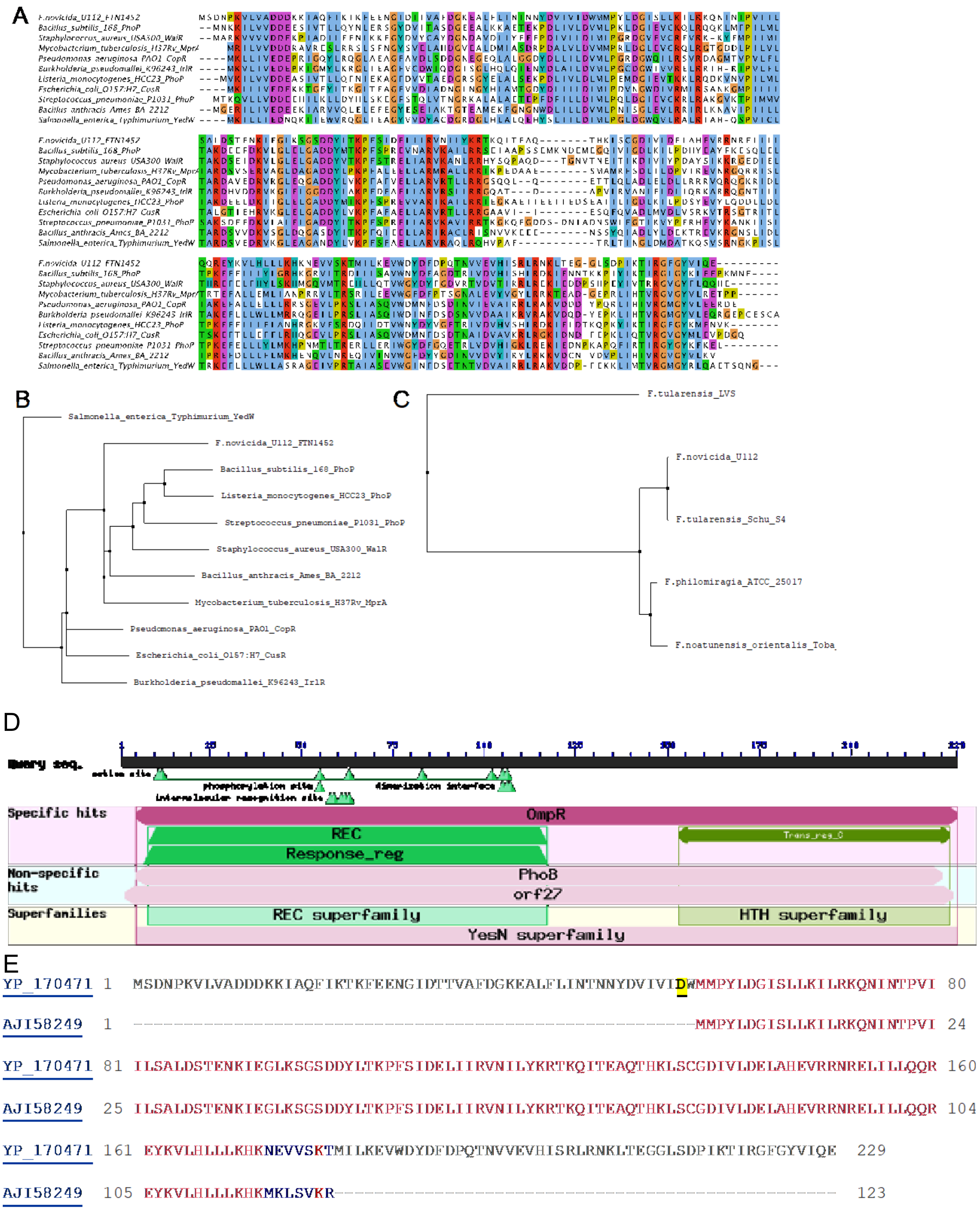

Figure S1. Sequence comparison of BfpR, BfpK and homologs. (A) Sequence alignment of BfpR and homologs, and (B) corresponding tree. Note that BfpR is not present in *Francisella tularensis* LVS. In *F. novicida*, two intact TCS systems are annotated FTN_1452-FTN_1453

(BfpR and its paired sensor kinase BfpK) and FTN_1715- FTN_1714 (KdpDE), one orphan sensor kinase FTN_1617 (QseC), and one orphan RR PmrA/QseB (FTN_1465). The TCS genes previously characterized in *F. tularensis* SchuS4 include two sensor kinases FTT_0094c (QseC) and FTT_1736c (KdpD) and two RRs: FTT_1543 (here named BfpR) and FTT_1557c (PmrA/QseB), all of which were considered to be "orphan" molecules as they are not paired in a normal TCS operon (RR plus sensor kinases). In *F. tularensis holarctica* strains, no intact TCS systems are annotated. This organism has two orphan Sensor kinases KdpD and QseC, and one orphan RR PmrA/QseB (FTL_0552). (C) Tree comparing BfpR homologs within *Francisella*. Analysis was done using JalView, where sequences were first aligned with Clustal Omega (Sievers et al., 2011), and compared using BLOSUM62 Neighbor Joining. (D) Conserved domains analysis of BfpR (gi|56708575|ref|YP_170471.1|) of *Francisella tularensis* subsp. *tularensis* SCHU S4. (https://www.ncbi.nlm.nih.gov/Structure/cdd/wrpsb.cgi?SEQUENCE=YP_170471.1&FULL. (E) *Francisella* LVS homolog alignment with BfpR. Alignment of YP_170471.1 (*F. tularensis* SchuS4 BfpR, identical to *F. novicida* BfpR) and AJI58249.1 (*F. holarctica* LVS). Predicted aspartate phosphorylation site D56 in BfpR highlighted in yellow. The LVS gene encodes only 123 of the 229 amino acids for the full-length protein. The first 56 and last 57 amino acids are missing, including the conserved aspartate at D56, which acts as the phosphoacceptor site. Since the aspartate is required for response regulator activation, this pseudogene is predicted to be inactive. Alignment was done using COBALT.

A

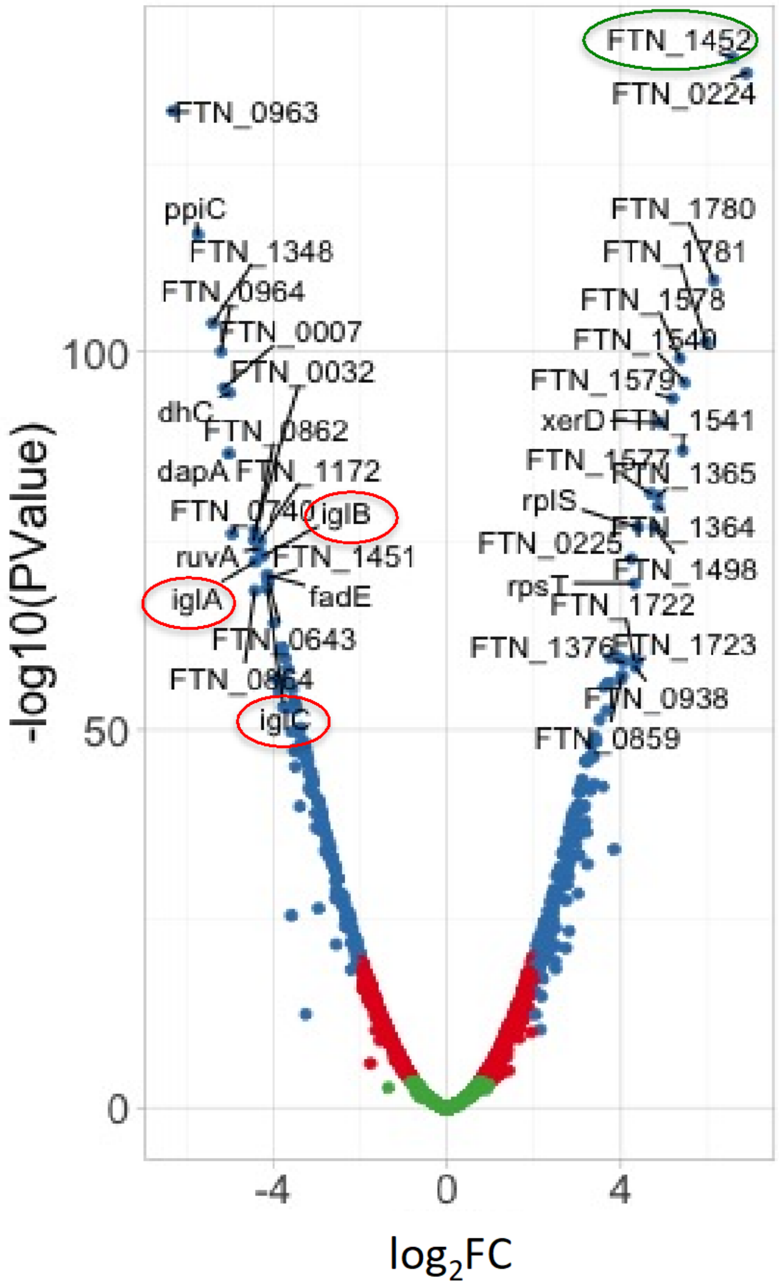
FTN_1452
FTN_0224
FTN_0963
ppiC
FTN_1348
FTN_0964
FTN_0007
FTN_0032
dhC
FTN_0862
dapA
FTN_1172
FTN_0740
iglB
ruvA
FTN_1451
iglA
fadE
FTN_0643
FTN_0864
iglC
FTN_1780
FTN_1781
FTN_1578
FTN_1540
FTN_1579
xerD
FTN_1541
FTN_1577
FTN_1365
rplS
FTN_1364
FTN_0225
FTN_1498
rpsT
FTN_1722
FTN_1376
FTN_1723
FTN_0938
FTN_0859
-log10(PValue)
log2FC
100
50
0
-4
0
4

B

TCS genes	BfpR vs BfpROX		WT vs BfpR		WT vs BfprOX	
Francisella novicida	FC	log10FC	FC	log10FC	FC	log10FC
**BfpR (FTN_1452)**	114.6	2.06	1.52	0.18	174.5	2.24
**BfpK (FTN_1453)**	4.66	0.67	1.16	0.07	5.41	0.73
**QseB (FTN_1465)**	0.50	-0.30	0.96	-0.017	0.48	-0.32
**QseC (FTN_1617)**	2.32	0.36	1.02	0.009	2.37	0.37
**kdpE (FTN_1714)**	2.54	0.40	0.77	-0.11	0.98	-0.008
**kdpD (FTN_1715)**	6.47	0.81	0.72	-0.14	1.84	0.26

C

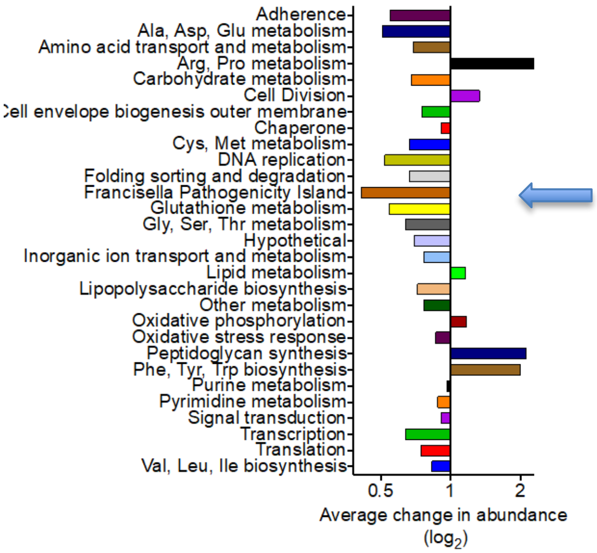

Figure S2. Transcriptional and proteomic profile of *bfpR*^{ox} vs. *bfpR.* (A) Volcano plot of RNASeq results of *bfpR*^{ox} vs. *bfpR* Tn-mutant. Blue dots have FDR<0.0005 and [log_2FC]>2, Red dots have false-discovery rate (FDR) <0.0005, Green dots did not change significantly [log_2FC]<2. *BfpR* (FNT_1452) expression is circled in green. Three genes from the Francisella Pathogenicity Island, *iglA*, *ilgB* and *iglC*, are circled in red. (B) Table of Changes of TCS genes in Francisella. The fold-change data from the full RNASeq data table was summarized to highlight the six TCS genes in Francisella. (C) Proteomics data showing fold change in abundance comparing *bfpR*^{ox} and *bfpR* mutant (log_2 scale) (Figure S5). The data suggest the overall broad impact of overexpressing *bfpR*. The arrow in (C) is pointing to the Francisella pathogenicity island protein cluster.

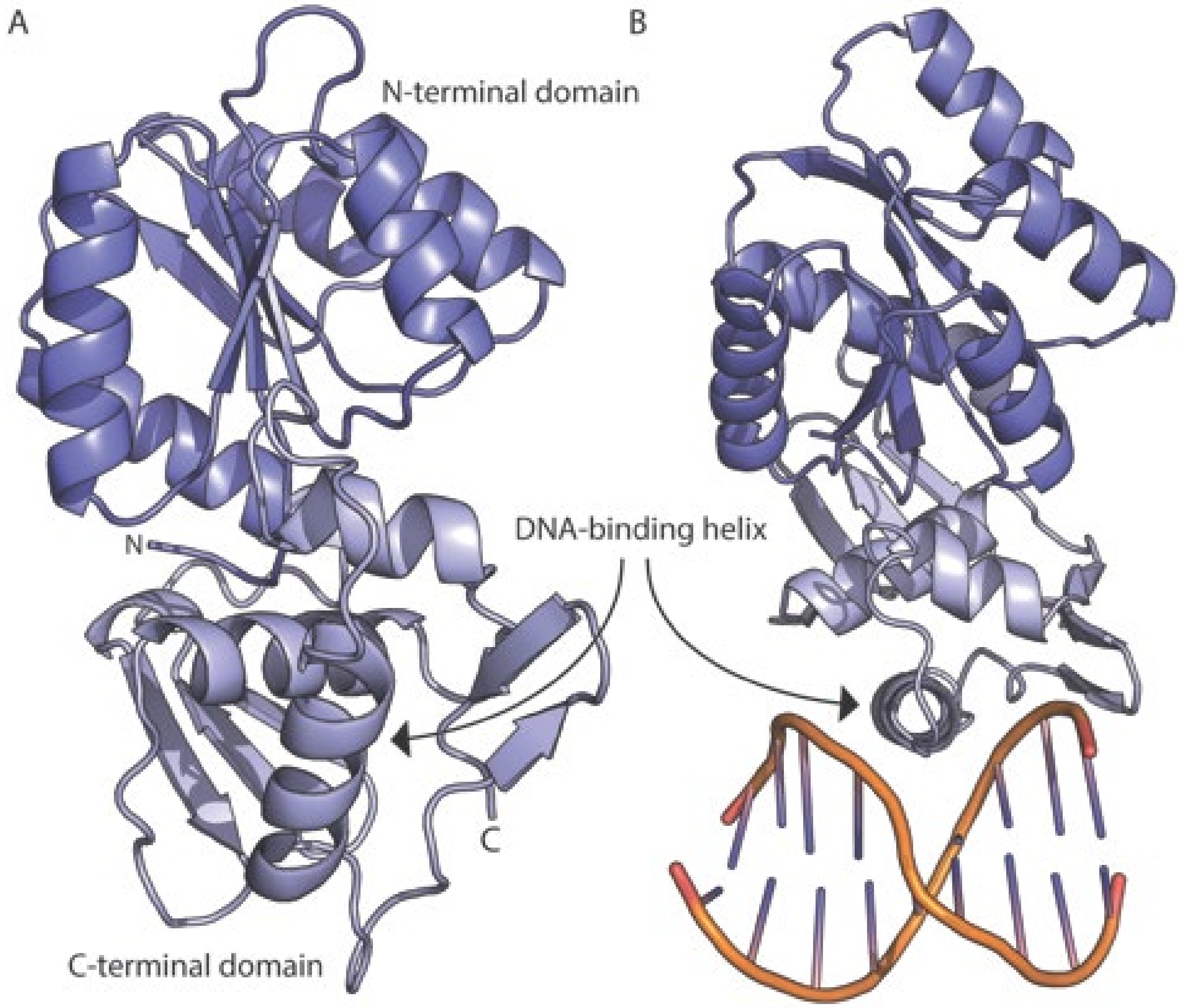

Figure S3. Full-length model of BfpR and *in silico* DNA binding. (A) A full-length model of BfpR was generated using the crystal structure of the receiver domain (dark blue) and building a homology model of the DNA binding domain (light blue). The model was built using MODELLER (Eswar et al., 2006) and refined in ICM-Pro (MolSoft LLC). (B) The full-length model was docked to a short DNA fragment, 5′-AACTGTTAC, which was generated using 3D-DART (van Dijk and Bovin, 2009). DNA docking was carried out using HADDOCK (van Zundert et al., 2016; Wassenaar et al. 2012). The top ranked docking pose accurately positioned the DNA-binding helix into the major groove of the DNA as expected based on the crystal structures of other DNA-bound response regulators.

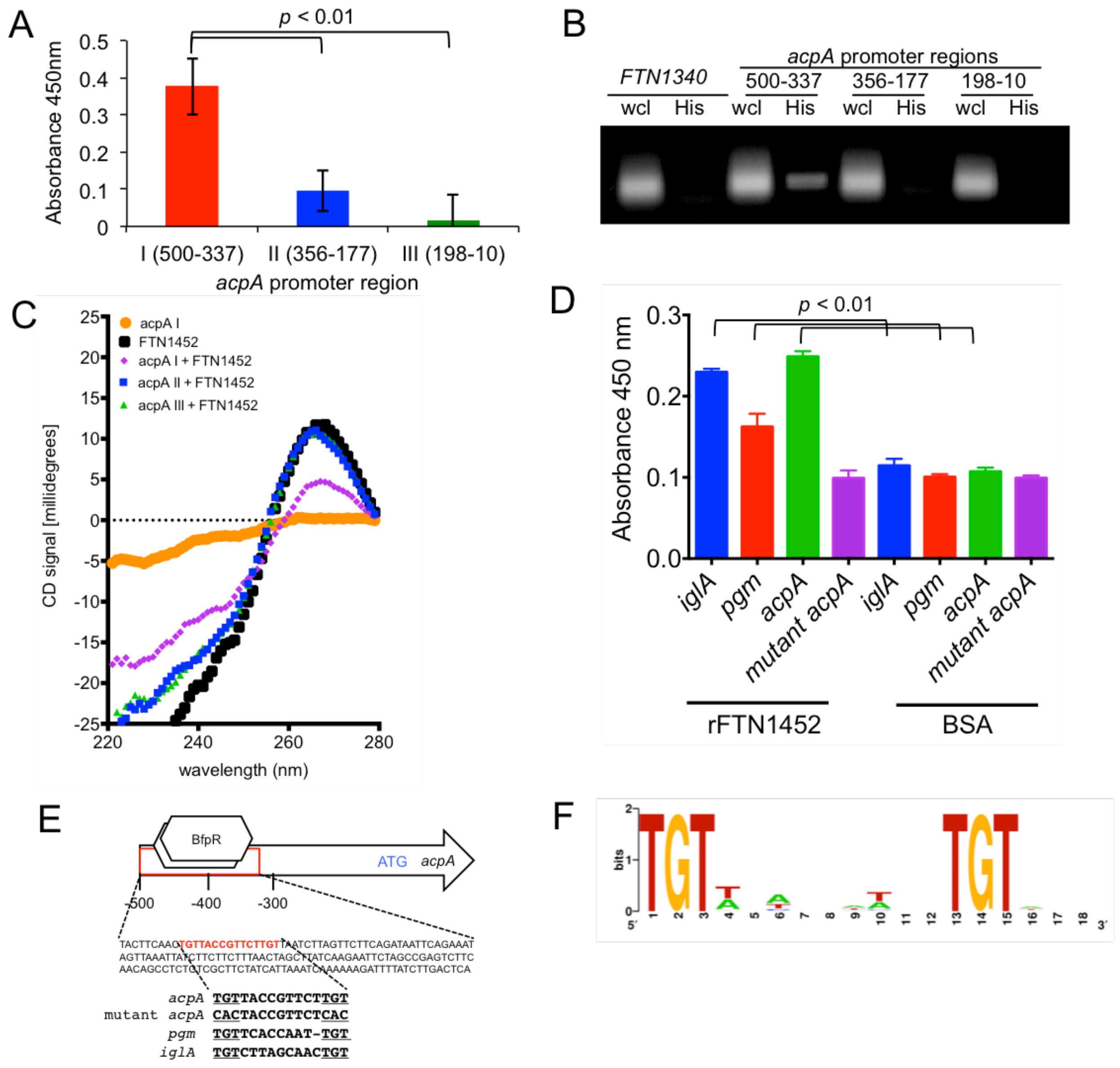

Figure S4: DNA binding of BfpR to distal region of *acpA* promoter. (A) ELISA showing binding of BfpR-His$_6$ to the *acpA* promoter regions: the most distal region (I, red), the middle region (II, blue), and proximal region (III, green). Binding was evaluated with BfpR-His$_6$ and without (coated with BSA). (B) ChIP-PCR from the BfpR-His$_6$ strain, where the three broad *acpA* promoter fragments were probed for binding. FTN1340 DNA was the negative control. (C) CD spectra were recorded from 220 nm to 280 nm in 2-nm steps on samples of BfpR-His$_6$ and the *acpA* promoter regions *acpA* I only (orange), BfpR-His$_6$ only (black), *acpA* I plus BfpR-His$_6$ (purple), and *acpA* II plus BfpR-His$_6$ (blue), and *acpA* III plus BfpR-His$_6$ (green). The changes observed are conformational changes due to interactions between DNA and protein. (D) Diagram showing the BfpR binding site in the *acpA* promoter and its alignment with *pgm* and *iglA*. (E) ELISA method showing binding of BfpR-His$_6$ to small *iglA, pgm, acpA* promoter fragments. Binding was evaluated with BfpR-His$_6$ and without. BfpR-His$_6$ binds to *iglA, pgm, acpA* small promoter fragments but not the mutated *acpA* fragment. (F) WebLogo of the putative binding site.

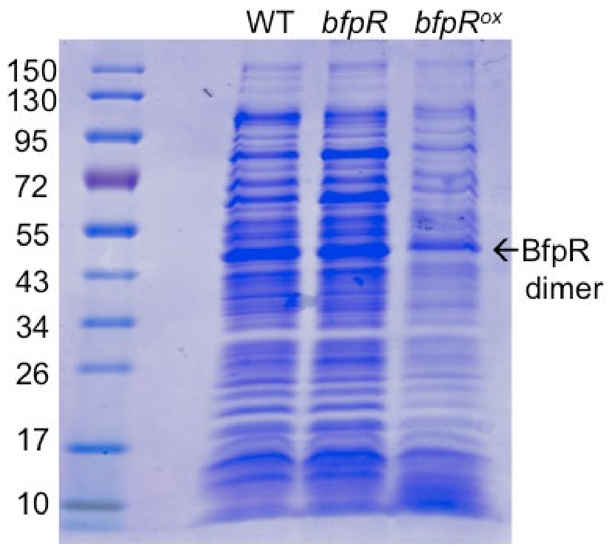

Figure S5. *bfpR* (FTN1452)-His_6 and *bfpR* overexpression. Lysates of WT, *bfpR,* and complemented overexpressing *bfpR*^{ox} from overnight culture run on Tris-Glycine gel then stained with Coomassie blue. The arrow indicates the most prominent band from the *bfpR*^{ox} lysate, at ~53 kDa, the size of the BfpR dimer.

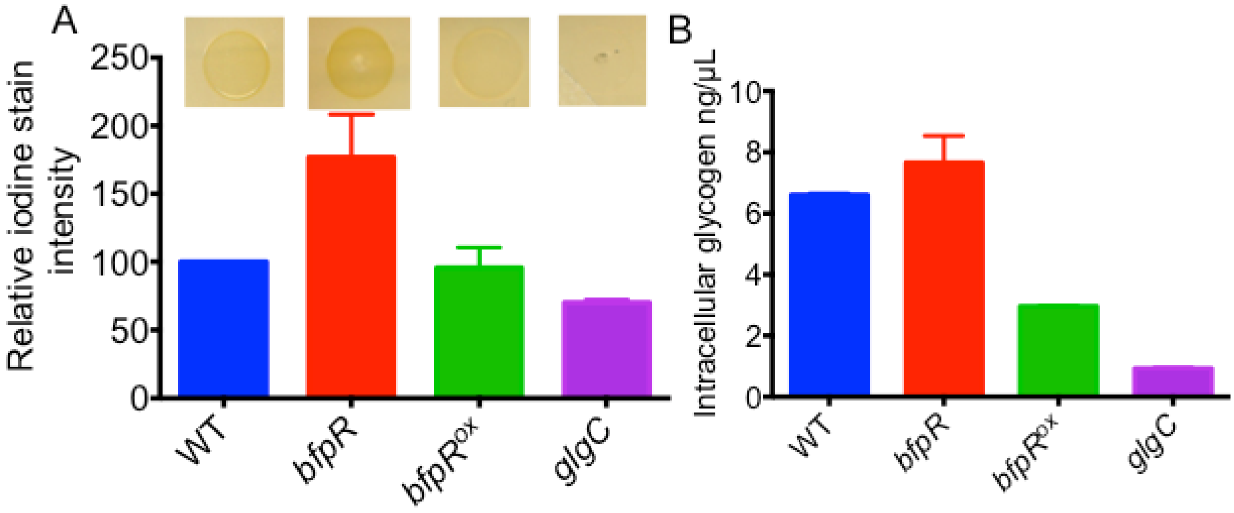

Figure S6. (A) Quantification of iodine staining intensity after 48 h at 37°C on TSBC agar with 0.5% glucose. *bfpR* produced more polysaccharide than WT, *bfpR*^{ox}, and *glgC*. Representative images shown above correspond to columns of the graph. (B) Intracellular glycogen quantification of WT, *bfpR*, *bfpR*^{ox} strain, and *glgC*. Data are shown as averages from three independent experiments, error bars indicate standard deviation, and significance was determined by Student's *t* test.

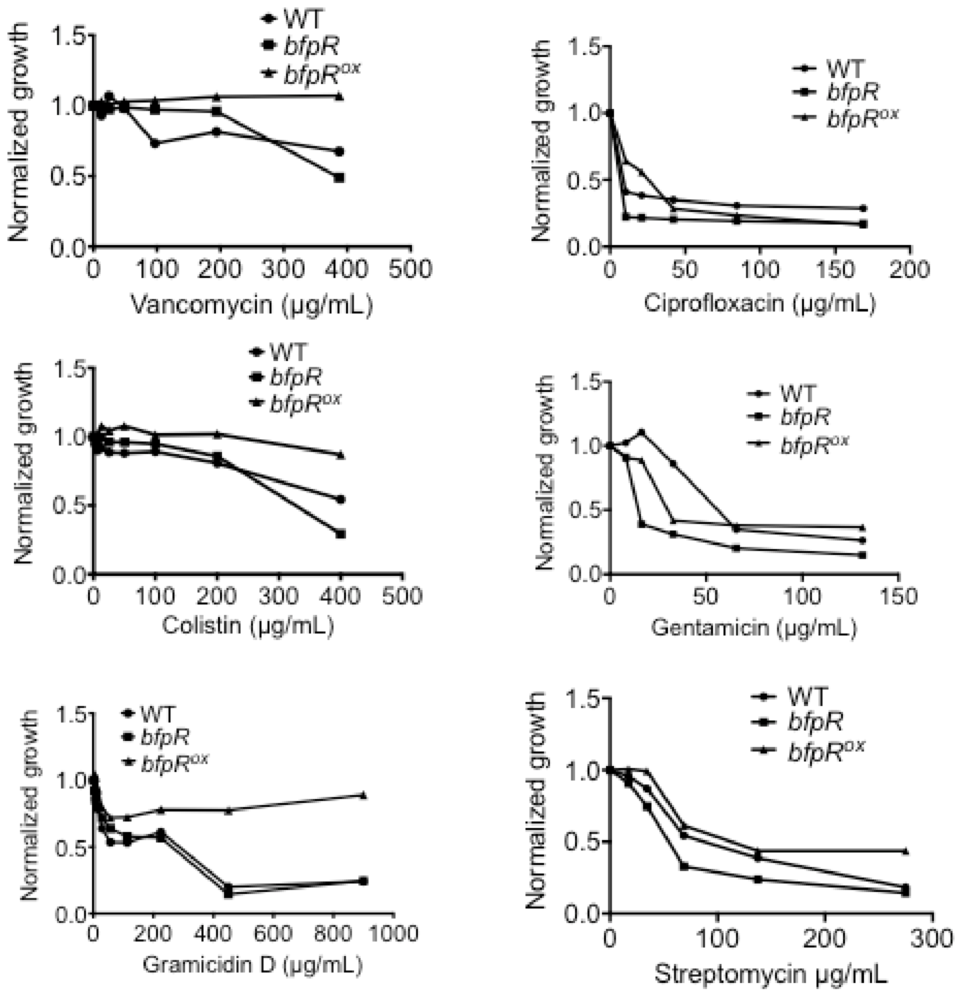

Figure S7. *bfpR* overexpression influences resistance to peptide antibiotics. (A) Survival of bacteria in TSBC with titration of peptide antibiotics. Antimicrobial assays of vancomycin, colistin, and gramicidin D against WT, *bfpR,* and *bfpR*^{ox}. The sensitivity to each was reduced for the *bfpR*^{ox} strain, while there were insignificant differences observed for WT and *bfpR.* (B) Antimicrobial assay of ciprofloxacin, gentamicin, and streptomycin against WT, *bfpR,* and *bfpR*^{ox} strains. No difference in sensitivity observed in any of the strains.

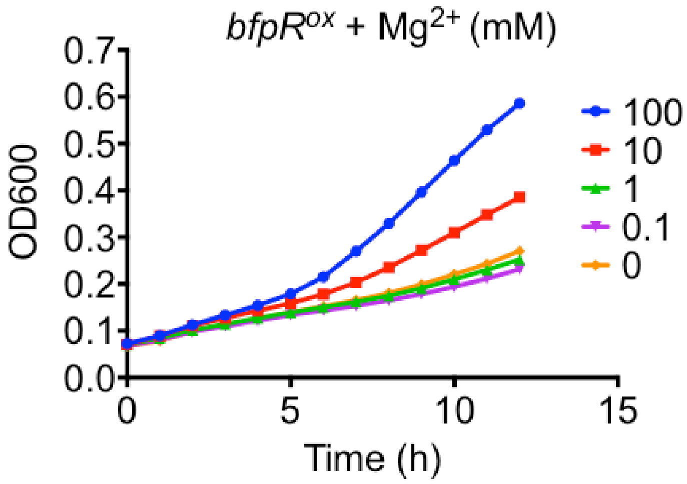

Figure S8. Concentration-dependent growth induction by Mg^{2+}. The growth rate of *bfpR*^{ox} in increasing Mg^{2+}, in TSBC. These results support the finding that the decrease growth rate of *bfpR*^{ox} is derepressed in high Mg^{2+}. This data shows that this effect is concentration dependent.

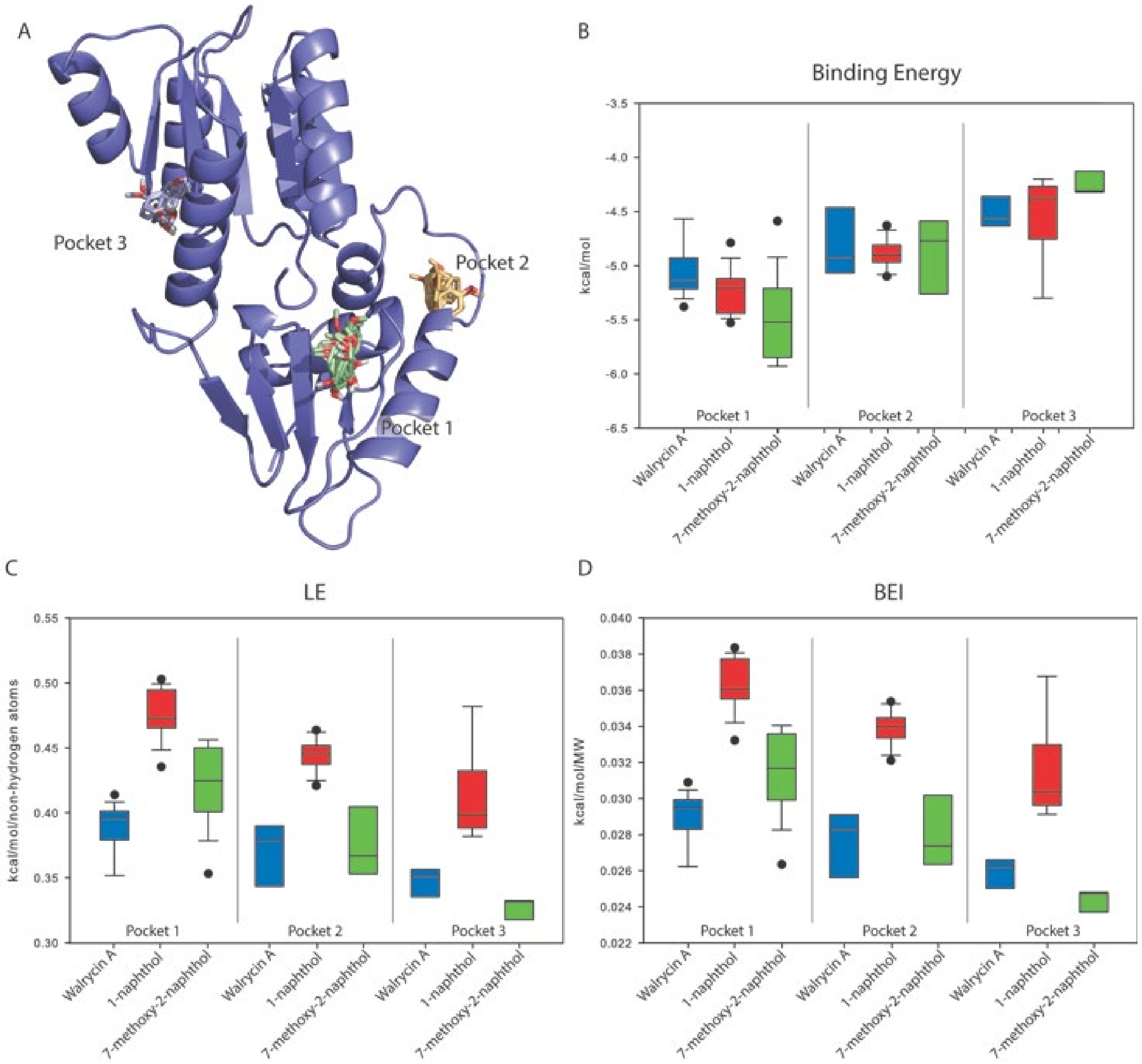

Figure S9. Small molecule docking to BfpR. (A) Top binding sites for docking walrycin A, 1-naphthol, and 7-methoxy-2-naphthol to our full-length model of BfpR localize to pockets 1 (green), 2 (yellow), and 3 (light blue). Pocket 2 is located at the end of the DNA-binding helix and Pocket 3 is in the dimerization interface. The top clusters were plotted by binding energy (B); ligand efficiency, LE, calculated by the kcal/mol/non-hydrogen atoms (C); and binding efficiency index, BEI, calculated by the kcal/mol/MW (D). These results indicate that each of the compounds has the potential to bind BfpR and that two of the most favored putative binding sites could directly impact BfpR DNA binding and dimerization activity.

Table S1. Primers and strains used in the study.

Name		Genotype, characteristics, or sequence	Source
Strains			
Francisella novicida U112		wildtype (WT), sequenced and annotated strain	ATCC
bfpR (FTN1452)		T20 (IS*Fn*2/FRT), KanR	ATCC
bfpR^{ox}		*FTN1452* Tn::*groEL-FTN1452, TetR*	This study
E. coli		One Shot® TOP10 Chemically Competent *E. coli*	Invitrogen
Plasmids			
Modified pKK214		pKK214 with EcoRI-PstI-XhoI-KpnI MCS added	This study
Primers			
FTN1452	forward	GGCTGCAGTTATTAGCTTAAAATTA	Invitrogen
FTN1452	reverse	ATGAATTCCTACTCTTGGATGACAT	Invitrogen
FTN1452_TOPO	forward	ATGAGTGACAACCCAAAAGTCTT	Invitrogen
FTN1452_TOPO	reverse	CTCTTGGATGACATAGCCAAAG	Invitrogen
acpA I (500-337)	forward	TACTTCAACTGTTACCGTT	Invitrogen
acpA I (500-337)	reverse	TCCGAGTCAAGATAAAATC (5'-biotinylation)	Invitrogen
acpA II (356-177)	forward	GATTTTATCTTGACTCGGA	Invitrogen
acpA II (356-177)	reverse	TTCGGTGTAAAAGCAATAT (5'-biotinylation)	Invitrogen
acpA III (198-10)	forward	ATATTGCTTTTACACCGAA	Invitrogen
acpA III (198-10)	reverse	TAGTTTGTTAGACTCAAAG (5'-biotinylation)	Invitrogen

Table S2: Data collection and refinement statistics (molecular replacement)

	1542N (6ONT)
**Data collection**	
Space group	$I4_132$
Cell dimensions	
a, *b*, *c* (Å)	128.726, 128.726, 128.726
α, β, γ (°)	90, 90, 90
Resolution (Å)	50.00–1.80 (1.83–1.80)[a]
R_{sym}	0.079 (0.740)
$I / \sigma I$	83.425 (2.205)
Completeness (%)	99.99 (99.94)
Redundancy	24.7 (8.0)
**Refinement**	
Resolution (Å)	45.51–1.80
No. reflections	17102
R_{work} / R_{free}	0.1824/0.2019
No. atoms	
Protein	969
Water	86
B-factors (Å^2)	
Protein	44.6
Water	52.8
R.m.s. deviations	
Bond lengths (Å)	0.005
Bond angles (°)	0.781

[a]Values in parenthesis correspond to the highest-resolution shell.

Table S3: RNASeq results for *F. novicida* Two-component system genes. FC = Fold Change. $Log_{10}FC$ = Log_{10} Fold Change (Shown on graphic in Supplemental Figure 2A). Fold Changes >2 fold up or down are highlighted in grey. As expected, overexpression of *bfpR* leads to significantly more *BfpR* RNA in the samples. Other TCS genes are also upregulated with that overexpression, shown in grey (>2 fold), with the exception of *qseB*. The transposon insertion mutant in *bfpR* does not show a significant alteration in expression of any of the TCS genes compared to the parental strain (WT).

	$BfpR^{OX}$ vs BfpR ($BfpR^{OX}$/ BfpR)		BfpR vs WT (BfpR/ WT)		$Bfpr^{OX}$ vs WT ($BfpR^{OX}$/ WT)	
	FC	$log_{10}FC$	FC	$log_{10}FC$	FC	$log_{10}FC$
**BfpR (FTN_1452)**	114.6	2.059	1.523	0.183	174.5	2.242
**BfpK (FTN_1453)**	4.657	0.668	1.162	0.065	5.414	0.733
**QseB (FTN_1465)**	0.496	-0.305	0.962	-0.017	0.477	-0.321
**QseC (FTN_1617)**	2.316	0.365	1.021	0.009	2.365	0.374
**KdpE (FTN_1714)**	2.536	0.404	0.774	-0.111	0.981	-0.008
**KdpD (FTN_1715)**	6.474	0.811	0.725	-0.140	1.839	0.264

Table S4: Full RNASeq data. Data Set 798 is the WT *F. novicida* sample (Actual Reads 12,547,978) vs. Set 801, *bfpR*, the Tn mutant of FTN1452 (Actual Reads 10,523,222; data sets from 2 runs; combined data sets provided).

Table S5: Full RNASeq data. Data Set 801 is *bfpR*, the Tn mutant of FTN1452 (Actual Reads 10,523,222; data sets from 2 runs; combined data sets provided) vs. Set 803 b*fpR*^{ox}, the overexpressing strain (Actual Reads 12,516,440). The file titles represent the comparisons being made in each set. For example, in 803 vs 801 comparison, 661 genes were down-regulated, 432 were non-differentially expressed and 688 were up-regulated comparing b*fpR*^{ox} to *bfpR* Tn mutant.

References

Eswar, N., Webb, B., Marti-Renom, M. A., Madhusudhan, M. S., Eramian, D., Shen, M. Y., Pieper, U., and Sali, A. (2006) Comparative protein structure modeling using Modeller. *Curr Protoc Bioinformatics* Chapter 5, Unit 5 6

Sievers F., Wilm A., Dineen D., Gibson T.J., Karplus K., Li W., Lopez R., McWilliam H., Remmert M., Söding J., Thompson J.D. and Higgins D.G. (2011) Fast, scalable generation of high-quality protein multiple sequence alignments using Clustal Omega. Mol. Syst. Biol. 7:539

van Dijk, M., and Bonvin, A. M. (2009) 3D-DART: a DNA structure modelling server. *Nucleic Acids Res* **37**, W235-239

van Zundert, G. C. P., Rodrigues, J., Trellet, M., Schmitz, C., Kastritis, P. L., Karaca, E., Melquiond, A. S. J., van Dijk, M., de Vries, S. J., and Bonvin, A. (2016) The HADDOCK2.2 Web Server: User-Friendly Integrative Modeling of Biomolecular Complexes. J Mol Biol 428, 720-725

Wassenaar, T. A., van Dijk, M., Loureiro-Ferreira, N., van der Schot, G., de Vries, S. J., Schmitz, C., van der Zwan, J., Boelens, R., Giachetti, A., Ferella, L., Rosato, A., Bertini, I., Herrmann, T., Jonker, H. R. A., Bagaria, A., Jaravine, V., Güntert, P., Schwalbe, H., Vranken, W. F., Doreleijers, J. F., Vriend, G., Vuister, G. W., Franke, D., Kikhney, A., Svergun, D. I., Fogh, R. H., Ionides, J., Laue, E. D., Spronk, C., Jurkša, S., Verlato, M., Badoer, S., Dal Pra, S., Mazzucato, M., Frizziero, E., and Bonvin, A. M. J. J. (2012) WeNMR: Structural Biology on the Grid. Journal of Grid Computing 10, 743-767
